# Assessment of Tumor Heterogeneity, as Evidenced by Gene Expression Profiles, Pathway Activation, and Gene Copy Number, in Patients with Multifocal Invasive Lobular Breast Tumors

**DOI:** 10.1371/journal.pone.0153411

**Published:** 2016-04-14

**Authors:** Nadine Norton, Pooja P. Advani, Daniel J. Serie, Xochiquetzal J. Geiger, Brian M. Necela, Bianca C. Axenfeld, Jennifer M. Kachergus, Ryan W. Feathers, Jennifer M. Carr, Julia E. Crook, Alvaro Moreno-Aspitia, Panos Z. Anastasiadis, Edith A. Perez, E. Aubrey Thompson

**Affiliations:** 1 Department of Cancer Biology, Mayo Clinic, Jacksonville, Florida, United States of America; 2 Division of Hematology/Oncology, Mayo Clinic, Jacksonville, Florida, United States of America; 3 Department of Health Sciences Research, Mayo Clinic, Jacksonville, Florida, United States of America; 4 Department of Laboratory Medicine and Pathology, Mayo Clinic, Jacksonville, Florida, United States of America; Baylor College of Medicine, UNITED STATES

## Abstract

**Background:**

Invasive lobular carcinoma (ILC) comprises approximately ~10–20% of breast cancers. In general, multifocal/multicentric (MF/MC) breast cancer has been associated with an increased rate of regional lymph node metastases. Tumor heterogeneity between foci represents a largely unstudied source of genomic variation in those rare patients with MF/MC ILC.

**Methods:**

We characterized gene expression and copy number in 2 or more foci from 11 patients with MF/MC ILC (all ER+, HER2-) and adjacent normal tissue. RNA and DNA were extracted from 3x1.5mm cores from all foci. Gene expression (730 genes) and copy number (80 genes) were measured using Nanostring PanCancer and Cancer CNV panels. Linear mixed models were employed to compare expression in tumor versus normal samples from the same patient, and to assess heterogeneity (variability) in expression among multiple ILC within an individual.

**Results:**

35 and 34 genes were upregulated (FC>2) and down-regulated (FC<0.5) respectively in ILC tumor relative to adjacent normal tissue, q<0.05. 9/34 down-regulated genes (*FIGF*, *RELN*, *PROM1*, *SFRP1*, *MMP7*, *NTRK2*, *LAMB3*, *SPRY2*, *KIT*) had changes larger than *CDH1*, a hallmark of ILC. Copy number changes in these patients were relatively few but consistent across foci within each patient. Amplification of three genes (*CCND1*, *FADD*, *ORAOV1*) at 11q13.3 was present in 2/11 patients in both foci. We observed significant evidence of within-patient between-foci variability (heterogeneity) in gene expression for 466 genes (p<0.05 with FDR 8%), including *CDH1*, *FIGF*, *RELN*, *SFRP1*, *MMP7*, *NTRK2*, *LAMB3*, *SPRY2* and *KIT*.

**Conclusions:**

There was substantial variation in gene expression between ILC foci within patients, including known markers of ILC, suggesting an additional level of complexity that should be addressed.

## Introduction

Invasive lobular carcinoma (ILC) accounts for approximately 8% to 14% of all breast cancers with a predilection for multifocal (MF) and multicentric (MC) distribution and for bilaterality [1]. The largest study of multiple breast lesions (n = 8935, including lobular and ductal histology), reported the incidence of MF carcinoma, defined as the presence of multiple tumors within the same quadrant of the same breast, at 15.5% [2]. The same study reported the incidence of multicentric (MC) carcinoma, defined as multiple tumors in different quadrants of the same breast as 5.2%. Given the lower frequency of ILC compared to invasive ductal carcinoma (IDC) and the lower frequency of MF/MC compared to unifocal breast cancer, there is a dearth of data on molecular/genomic aspects of MF ILC specifically, with most studies focusing on either MF/MC versus unifocal carcinoma or lobular versus ductal histology. A study of 812 patients with ipsilateral invasive breast cancer reported 7.6% patients with lobular histology, 17.4% MF/MC and 2.1% (17/812) of patients with MF/MC ILC [3]. Additionally, the origin of MF breast cancer is unclear, plausible explanations include intramammary spread from a single primary tumor or alternatively, tumors arising from separate progenitor cells [4].

The characteristics and lack of molecular data in MF and ILC subtypes leads to a conundrum: The vast majority of ILCs are of lower histological grade, express estrogen receptor (ER), lack HER2 overexpression/gene amplification, and fall into the ‘luminal’ molecular subgroup, [1, 5, 6] characteristics associated with higher survival rates and relatively low recurrence rates [7–10]. Conversely, MF breast cancer is associated with an increased risk of regional lymph node metastases, increased risk of local relapse and worse outcome [11–14]. Stratification by intrinsic molecular subtyping in a study of 444 consecutive invasive breast cancer patients, showed that within the luminal A subtype (associated with higher survival rates), multifocal luminal A patients (n = 79) had significantly worse survival than unifocal luminal A (n = 212) patients and multifocal luminal B patients (n = 13) had significantly worse survival than unifocal luminal B patients (n = 29) [15]. The survival analyses were not further stratified for lobular and ductal histology within the luminal subgroups.

In this study we provide a high level molecular characterization of multiple foci from eleven patients with ER-positive, HER2-negative MF/MC ILC, herein referred to as MF ILC. Using a gene expression panel of 730 known cancer genes (606 genes from 13 canonical cancer pathways and 124 cancer associated driver genes), and a gene copy number panel of 80 known cancer genes, we examined the extent to which the genomic architecture varies between multiple foci in patients with ILC and between MF ILC and adjacent normal tissue at the level of individual genes and pathways. Our study design included multiple punches from each focus. This design allowed us to examine gene expression and gene copy number in this rare group of patients at the level of differences between ILC foci and adjacent normal tissue; intra-tumor heterogeneity; and the overall level of tumor-tumor variability (heterogeneity) within a patient relative to variability between tumors from different patients that is not attributable to intra-tumor variability.

## Methods

### Patient population

Genomic profiling was performed on eleven patients with invasive lobular MF breast cancer. Three x 1.5mm cores were punched from FFPE blocks of each primary focus by an experienced breast pathologist (XG), who selected areas that were >70% tumor cells in order to minimize the potential effect of differences in tumor versus stroma content. For patient 1, cores were punched from two lobular multifocal lesions in the right breast and one lobular unifocal lesion in the left breast; for all other patients the multiple lesions were in the same breast. 1.5mm cores were available from adjacent normal breast tissue in seven of the eleven multifocal patients and normal breast tissue from an additional six patients with other breast cancer subtypes.

All 11 patients and their foci were positive for ER and negative for HER2 by immunohistochemistry (IHC), while three patients were discrepant for PR status (Table 1). All tumors were confirmed by our pathologist (XG) as ILC pathology. All tumors with the exception of one were IHC negative for E-cadherin. One tumor (patient 7, tumor 1 was weakly positive for E-cadherin, detailed in S1 Fig). Patient material and clinical characteristics are summarized in Table 1.

**Table 1:** Patient material and clinical characteristics.

Patient	Age/Sex/Race, Primary surgical treatment	Focus size/cm	Distance between foci/cm	ER	PR	HER2	E-cad	Location	Histology/subtype	TNM stage	Final Stage	Grade	ALND, # pos LN	Genomic data	Adjacent normal available
1	44 / F / White	4.0	>4.0	+	+	-	-	Right UOQ	Lobular/classic	T2 N3a M0	IIIC	1	yes, right, 23/25	yes	yes
	R MRM and L total mastectomy	1.8		+	-	-	-	Right LOQ	Lobular/classic	T2 N3a M0		1		yes	
		1.0		+	+	-		Left UOQ	Lobular/classic	T1b N0 M0		1		yes	
															
2	61 / F / White	3.5	2.7	+	-	-	-	Right UOQ	Lobular/pleomorphic	T2 N2a M0	IIIC	2	yes, 7/18	yes	yes
	R mastectomy with ALND	1.5		+	+	-	-	Right UOQ	Lobular/classic	T2 N2a M0		1		yes	
		1.0						Right UOQ	Lobular/classic	T2 N2a M0		1		no	
		0.5						Right UOQ	Lobular/classic	T2 N2a M0		1		no	
															
3	66 / F / White	5.0	1.4	+	+	-	-	Right UOQ/midline	Lobular/pleomorphic	T2 N1a M0	IIB	2	yes, 1/25	yes	
	R mastectomy with ALND	1.4		+	+	-	-	Right UIQ	Lobular/classic	T2 N1a M0		1		yes	
															
4	87 / F / White	6.5	>3.0	+	+	-	-	Right UOQ	Lobular/alveolar	T3 N1a M0	IIIA	2	yes, 1/23	yes	yes
	R mastectomy with ALND	1.4		+	+	-	-	Right LOQ	Lobular/classic	T3 N1a M0		1		yes	
															
5	66 / F / White	2.5	3.5	+	+	-	-	Right UOQ	Lobular/pleomorphic	T2 N0 M0	IIA	2	no	yes	
	R lumpectomy and SLNB	2.2		+	+	-	-	Right UOQ	Lobular/pleomorphic	T2 N0 M0		2		yes	
															
6	56 / F / White	2.0	3.0	+	+	-	-	Right UOQ	Lobular/classic	T1c N0 M0	IA	1	no	yes	yes
	R mastectomy and SLNB	1.2		+	+	-	-	Right UOQ	Lobular/classic	T1c N0 M0		1		yes	
															
7	65 / F / White	1.2	11.0	+	+	-	+*	Right UIQ	Lobular/trabecular	T1c N0 M0	IA	1	no	yes	yes
	bilateral mastectomy and SLNB	0.8		+	+	-	-	Right UOQ	Lobular/classic	T1c N0 M0		1		yes	
															
8	68 / F / White	2.0	13.0	+	-	-	-	Left LIQ	Lobular/classic	T1c N1a M0	IIA	1	yes, 2/10	yes	
	L mastectomy with ALND	1.5		+	+	-	-	Left UOQ	Lobular/classic	T1c N1a M0		1		yes	
															
9	67 / F / White	3.2	2.5	+	+	-	-	Left UIQ	Lobular/pleomorphic	T2 N3a M0	IIIC	2	yes, 11/33	yes	
	L mastectomy with ALND	1.0		+	+	-	-	Left UIQ	Lobular/classic	T2 N0 M0		1		yes	
															
10	60 / F / White	6.0	3.0	+	+	-	-	Right LIQ	Lobular/pleomorphic	T3 N3a M0	IIIC	2	yes, 14/24	yes	yes
	R mastectomy with ALND	0.7		+	+	-	-	Right LOQ	Lobular/pleomorphic	T3 N3a M0		2		yes	
		0.3						Right UOQ	Lobular/pleomorphic	T3 N3a M0		2		no	
															
11	73 / F / White	4.5	1.5	+	+	-	-	UI and UOQ	Lobular/pleomorphic	T2 N1a M0	IIB	2	yes, 2/24	yes	yes
	R mastectomy with ALND	0.6		+	+	-	-	Right central	Lobular/pleomorphic	T2 N1a M0		2		yes	

Abbreviations: MRM, modified radical mastectomy; UOQ, Upper outer quadrant; UIQ, upper inner quadrant; LOQ, left outer quadrant; ALND, Axillary lymph node dissection; SLNB, Sentinel lymph node biopsy.

* Weakly positive. This tumor was dominant classic lobular, with some mixed trabecular pattern.

#### Ethics Statement

All breast tumor samples were collected between 2012 and 2014 according to a protocol that was reviewed and approved by the Mayo Clinic Institutional Review Board under protocol 13–009696. The review board approved waiver of the requirement to obtain informed consent in accordance with 45 CFR 46.116. Patient records/information were anonymized and de-identified prior to analysis.

### RNA and DNA extraction

RNA and DNA were extracted from each 1.5mm punch with the Qiagen AllPrep FFPE kit as per the manufacturer’s instructions, with the exception that proteinase K digestion times at 65°C were extended to overnight, as previously described [16].

### Gene expression data

Gene expression on the NanoString® platform was assessed with the NanoString PanCancer Pathways panel of 730 genes designed to capture the activity of thirteen canonical hallmarks of cancer pathways (606 pathway genes: Notch, Wnt, Hedgehog, TGFB, MAPK, STAT, PI3K, RAS, Chromatin modification, transcriptional regulation, DNA damage control, cell cycle and apoptosis), 124 cancer associated driver genes and 40 reference genes. 100ng of each total RNA sample was prepared as per the manufacturer’s instructions under the high sensitivity protocol. Gene expression was quantified on the NanoString nCounter^TM^ and raw counts were generated with nSolver^TM^. Raw counts were normalized against 36/40 reference genes, selected to have the least variance with the geNorm algorithm [17].

Normalized gene counts and raw data (RCC) files are available at GEO (accession number GSE79058).

### Gene copy number data

Gene copy number on the NanoString platform was assessed with the NanoString Cancer CNV panel (version 1, average three probes/gene). 500ng of DNA were prepared as per the manufacturer’s instructions (Alu digest and high sensitivity protocols). Copy number was calculated within the nSolver software (NanoString). Briefly, the NanoString Cancer CNV panel contains 2–3 probes in each of 80 known cancer genes and 54 invariant control probes used for normalization. Copy number was estimated as twice the ratio of the average probe count per gene in each patient to the average probe count per gene in a set of 16 adjacent normal punches extracted with the same protocol as the multifocal tumors (seven of which were from the multifocal ILC patients marked in Table 1 and nine punches from adjacent normal waste tissue of patients with other types of breast cancer).

*CCND1* amplification identified on the NanoString platform was validated with quantitative PCR (qPCR). Reactions were performed in duplicate with 20ng gDNA, TaqMan Universal PCR master mix, RNase P primer/ probe (4403328), and the *CCND1* primer/probe set (Life Technologies). Amplification data were collected with an Applied Biosystems Viia7 sequence detector and analyzed with ViiA 7 RUO software. CT values were normalized to control RNase P, and abundance was calculated using the ΔΔCT method [18]. Copy number gains in individual tumor samples were calculated relative to copy number in adjacent normal tissue from the same patient.

### Statistical analyses

#### Differential gene expression and heterogeneity analysis

Linear mixed models were used to assess between-tumor heterogeneity and difference in expression between foci and adjacent normal tissue. Normalized gene expression on the log (base 2) scale was the response variable. All models included both patient and tumor random effects. Tumor heterogeneity analyses included tumor grade (which correlated with lobular subtype: classic, pleomorphic, trabecular or alveolar) as an additional fixed effect. Fold change was estimated for the comparison of tumor versus adjacent normal analyses.

When conducting the heterogeneity analyses, the restricted maximum likelihood approach was used for model fitting to allow estimation of three standard deviation parameters for the three sources of variability: patient (σ_P_), foci (σ_F_), and punch (σ_IT_). Using data from three punches for each tumor, we define the intra-tumor variability, as HET.IT = σ_IT_^2^/ (σ_IT_^2^+ σ_F_^2^+ σ_P_^2^) × 100%. We define the within-patient between-foci heterogeneity, as HET.F = σ_t_^2^/ (σ_IT_^2^+ σ_F_^2^+ σ_P_^2^) × 100%. This is the variation among foci from the same patient (σ_F_^2^) expressed as a percentage of the total variation: the sum of the intra-tumor variability (σ_IT_), the within-patient variability (σ_F_^2^) and the patient to patient variability (σ_P_^2^). Similarly, we define the between patient heterogeneity, as HET.P = σ_P_^2^/ (σ_IT_^2^+ σ_F_^2^+ σ_P_^2^) × 100%.

Likelihood ratio tests were used to test the null hypothesis, H_0_: HET.F = 0, or equivalently H_0_: σ_F_ = 0. Two approaches were used to account for multiple testing for all of these analyses: Holm adjusted p-values,[19] and false discovery rate (FDR) estimates, q-values, obtained via the Benjamini-Hochberg approach.[20] Statistical analyses were conducted with R version 3.0.2.

#### Differential gene pathway analysis

Pathway dysregulation was scored for each focus in thirteen canonical cancer pathways[21] within the NSolver software (NanoString) using Principal Component (PC) analysis with adjacent normal tissue as the baseline reference. Data for each pathway were scaled before taking the first PC by dividing each gene’s log2 expression values by the greater of either their standard deviation or 0.05. Using the pathway scores calculated in nSolver, we performed differential expression analysis using the same regression model as in the gene-level differential expression analysis. These regressions were used to calculate a p-value for the association of each pathway of tumor versus adjacent normal tissue. Global significance statistics were also calculated for each pathway by measuring the cumulative evidence for the differential expression of genes in a pathway. For MF ILC tumors, global significance of each pathway was calculated as the square root of the pathway’s average squared t-statistic. Global significance for each pathway was then plotted against linear association pathway scores.

## Results

### Differential Gene Expression between ILC tumors and adjacent normal tissue

Several studies report worse outcome of multifocal relative to unifocal breast cancer, [11–15] and some evidence (although limited) suggests that multifocal breast cancer results from intramammary spread from a single primary tumor [22–25]. Under this scenario, we hypothesized that potential prognostic markers of MF ILC would be common to both foci, in which case they could be identified by differential expression analyses between all tumors within our MF ILC sample set and adjacent normal tissue. Hence, using the Nanostring PanCancer pathways panel, we first compared gene expression of 730 known cancer genes in multiple ILC foci of 11 patients to adjacent normal tissue from 7 of those patients.

Amongst the 730 genes tested, (S1 Table) there was evidence of differential expression (unadjusted p<0.05) in tumor relative to adjacent normal tissue for 253 genes with an estimated false discovery rate (FDR) of 14%. The expression in ILC tissue was estimated to be two or more fold that of adjacent normal tissue for 73 of the 253 genes, and to be less than half (FC<0.5) that of expression in normal tissue for 60 of these genes. 34 genes were significantly down-regulated (FC<0.5) with q<0.05, nine of which (*FIGF*, *RELN*, *PROM1*, *SFRP1*, *MMP7*, *NTRK2*, *LAMB3*, *SPRY2*, *KIT)* showed larger fold changes (0.06 to 0.26) than *CDH1* (0.28), which encodes for E-cadherin, the loss of which is a hallmark of ILC. *COL11A1* and *PKMYT1* showed the highest fold change of genes that were significantly up-regulated (with q<0.05) with FC of 25.0 and 11.7 respectively. Differentially expressed genes with FC>2.0 or 0.5 and q<0.05 are shown in Table 2. Results for all 730 genes are shown in S1 Table.

**Table 2:** Differential gene expression between MF ILC and adjacent normal tissue.

Gene	FC	Unadjusted P-value	Adjusted P-value	qval (FDR)	Tumor expression
COL11A1	24.99	1.33E-03	8.97E-01	1.71E-02	↑
PKMYT1	11.66	2.30E-05	1.67E-02	2.79E-03	↑
COMP	9.23	2.35E-03	1.00E+00	2.29E-02	↑
COL1A1	8.40	2.96E-05	2.14E-02	3.08E-03	↑
SIX1	7.93	2.15E-03	1.00E+00	2.20E-02	↑
BMP8A	6.94	2.01E-03	1.00E+00	2.16E-02	↑
ZIC2	5.47	4.55E-03	1.00E+00	3.46E-02	↑
CCNE2	5.05	6.68E-03	1.00E+00	4.47E-02	↑
UBE2T	5.02	2.04E-05	1.48E-02	2.79E-03	↑
MCM2	4.56	2.74E-04	1.93E-01	7.40E-03	↑
COL3A1	4.50	5.76E-05	4.16E-02	4.67E-03	↑
FN1	4.36	2.36E-04	1.67E-01	7.11E-03	↑
CREB3L1	4.30	2.43E-06	1.77E-03	8.87E-04	↑
LEF1	4.19	9.45E-04	6.46E-01	1.47E-02	↑
INHBA	4.14	2.07E-03	1.00E+00	2.19E-02	↑
CDKN2A	4.08	4.68E-03	1.00E+00	3.52E-02	↑
COL5A2	3.92	1.50E-05	1.09E-02	2.73E-03	↑
E2F1	3.68	2.19E-04	1.56E-01	7.11E-03	↑
COL1A2	3.58	4.26E-04	2.97E-01	9.26E-03	↑
GATA3	3.50	2.25E-04	1.59E-01	7.11E-03	↑
COL5A1	3.37	1.58E-04	1.13E-01	6.97E-03	↑
CACNA1D	3.05	2.58E-04	1.82E-01	7.23E-03	↑
IL20RB	3.00	6.78E-04	4.66E-01	1.15E-02	↑
CDKN2B	2.92	6.62E-05	4.78E-02	4.84E-03	↑
CBLC	2.91	3.58E-03	1.00E+00	2.90E-02	↑
HIST1H3H	2.76	1.62E-04	1.16E-01	6.97E-03	↑
BRIP1	2.49	3.04E-03	1.00E+00	2.64E-02	↑
PAX8	2.48	6.26E-03	1.00E+00	4.27E-02	↑
POLE2	2.43	1.08E-05	7.90E-03	2.64E-03	↑
TGFB3	2.41	1.01E-04	7.22E-02	5.00E-03	↑
FEN1	2.38	1.33E-03	8.98E-01	1.71E-02	↑
MAPT	2.32	1.32E-03	8.90E-01	1.71E-02	↑
CCND1	2.28	3.43E-04	2.40E-01	8.35E-03	↑
EZH2	2.18	7.25E-04	4.98E-01	1.19E-02	↑
CREB3L4	2.07	3.17E-03	1.00E+00	2.68E-02	↑
FIGF	0.06	1.50E-03	1.00E+00	1.80E-02	↓
RELN	0.12	4.05E-03	1.00E+00	3.11E-02	↓
PROM1	0.13	1.40E-03	9.44E-01	1.74E-02	↓
SFRP1	0.15	5.02E-03	1.00E+00	3.66E-02	↓
MMP7	0.17	1.52E-03	1.00E+00	1.80E-02	↓
NTRK2	0.22	3.62E-04	2.53E-01	8.52E-03	↓
LAMB3	0.23	2.24E-03	1.00E+00	2.22E-02	↓
SPRY2	0.24	2.13E-04	1.51E-01	7.11E-03	↓
KIT	0.26	2.17E-04	1.54E-01	7.11E-03	↓
CDH1	0.28	4.57E-04	3.17E-01	9.26E-03	↓
IL22RA1	0.29	4.86E-03	1.00E+00	3.59E-02	↓
LIFR	0.29	4.47E-04	3.11E-01	9.26E-03	↓
MET	0.30	5.16E-04	3.57E-01	9.90E-03	↓
EGFR	0.30	2.93E-04	2.06E-01	7.63E-03	↓
FGF10	0.31	1.07E-03	7.31E-01	1.58E-02	↓
ITGB4	0.31	8.07E-05	5.81E-02	5.00E-03	↓
FLNC	0.31	5.65E-03	1.00E+00	3.97E-02	↓
PAK3	0.32	7.08E-03	1.00E+00	4.66E-02	↓
ITGB3	0.35	1.66E-03	1.00E+00	1.90E-02	↓
PDGFRA	0.36	5.60E-04	3.87E-01	1.01E-02	↓
CACNB2	0.37	1.03E-04	7.36E-02	5.00E-03	↓
ITGB8	0.38	1.19E-03	8.05E-01	1.66E-02	↓
ZBTB16	0.38	1.78E-03	1.00E+00	2.00E-02	↓
FZD7	0.38	1.72E-04	1.23E-01	6.99E-03	↓
CREB5	0.41	1.22E-03	8.27E-01	1.68E-02	↓
KLF4	0.41	2.17E-03	1.00E+00	2.20E-02	↓
TCF7L1	0.43	5.59E-04	3.87E-01	1.01E-02	↓
MAML2	0.46	2.43E-04	1.72E-01	7.11E-03	↓
MYC	0.46	5.21E-03	1.00E+00	3.71E-02	↓
PLD1	0.47	4.84E-04	3.36E-01	9.55E-03	↓
GAS1	0.47	6.08E-03	1.00E+00	4.23E-02	↓
ITGB6	0.48	3.36E-04	2.36E-01	8.35E-03	↓
TGFBR2	0.48	6.75E-04	4.65E-01	1.15E-02	↓
CDC14A	0.50	3.32E-03	1.00E+00	2.75E-02	↓

Differential gene expression: ILC tumors versus adjacent normal tissue, FDR<0.05 and FC>2 or <0.5, ordered by direction and fold change. FC = fold change. FDR = false discovery rate.

### Differential Pathway Expression between ILC and adjacent normal tissue

**Fig 1:**
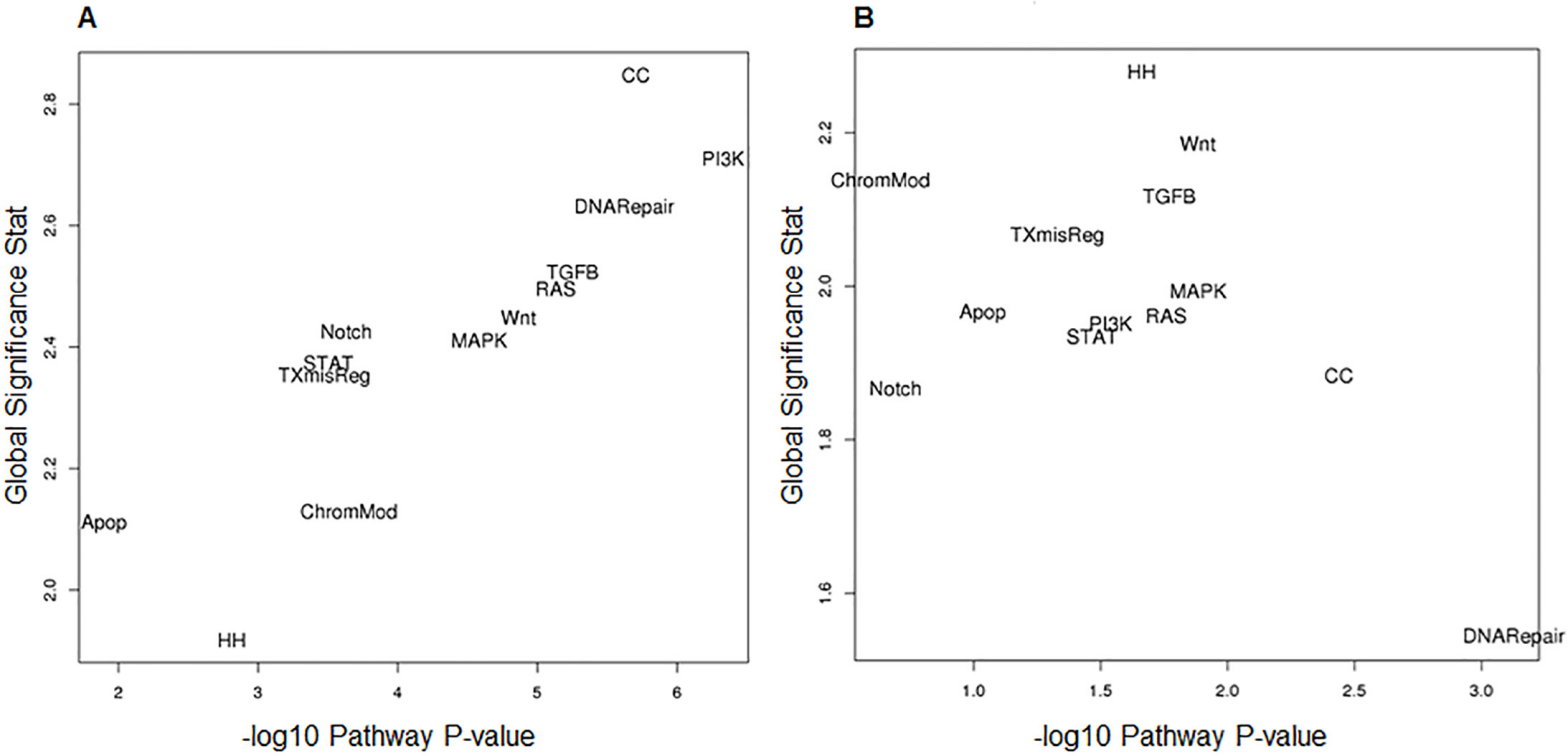
MF ILC pathway significance plots. A. Differential pathway expression in ILC tumor relative to adjacent normal tissue and B. Differential pathway expression between patient foci. Using the pathway scores, we performed differential expression analysis using the same regression model as in the gene-level differential expression analysis. These regressions were used to calculate a p-value for the association of each pathway of focus versus adjacent normal tissue (A) and between patient foci (B). Global significance statistics were calculated for each pathway by measuring the cumulative evidence for the differential expression of genes in a pathway relative to adjacent normal tissue (A) and between patient foci (B). Global significance for each pathway was then plotted against linear association pathway scores. There is agreement on both scales with the greatest difference in the PI3K and cell cycle pathways in ILC foci relative to adjacent normal tissue (A) and little difference in any pathway as measured between foci within patients (B).

We used two different statistical approaches to assess significance of 13 canonical cancer pathways in ILC tumors relative to normal adjacent tissue (Fig 1A). Firstly, we scored each sample for pathway dysregulation and performed differential expression analysis of these scores to measure association of each pathway in ILC tumor (plotted as–log10 p-value. Secondly, we used global significance statistics as a measure of the cumulative evidence for differential expression of genes in each pathway.

For the ILC tumors, the global significance score and pathway score p-value were in good agreement and all pathways were significantly different between ILC MF and adjacent normal tissue at p<0.05. The strongest associated pathways on both measures of association were PI3K and cell cycle, closely followed by DNA repair, TGFβ, RAS, Wnt and MAPK. Apoptosis and Hedgehog pathways were the least significant by both measures. The largest differences in absolute fold-change were PI3K (FC = 1098) and RAS (FC = 291) pathways (S2 Table).

### Differential genomic architecture of multiple foci within ILC patients

#### Gene copy number in multiple foci in ILC patients

**Fig 2:**
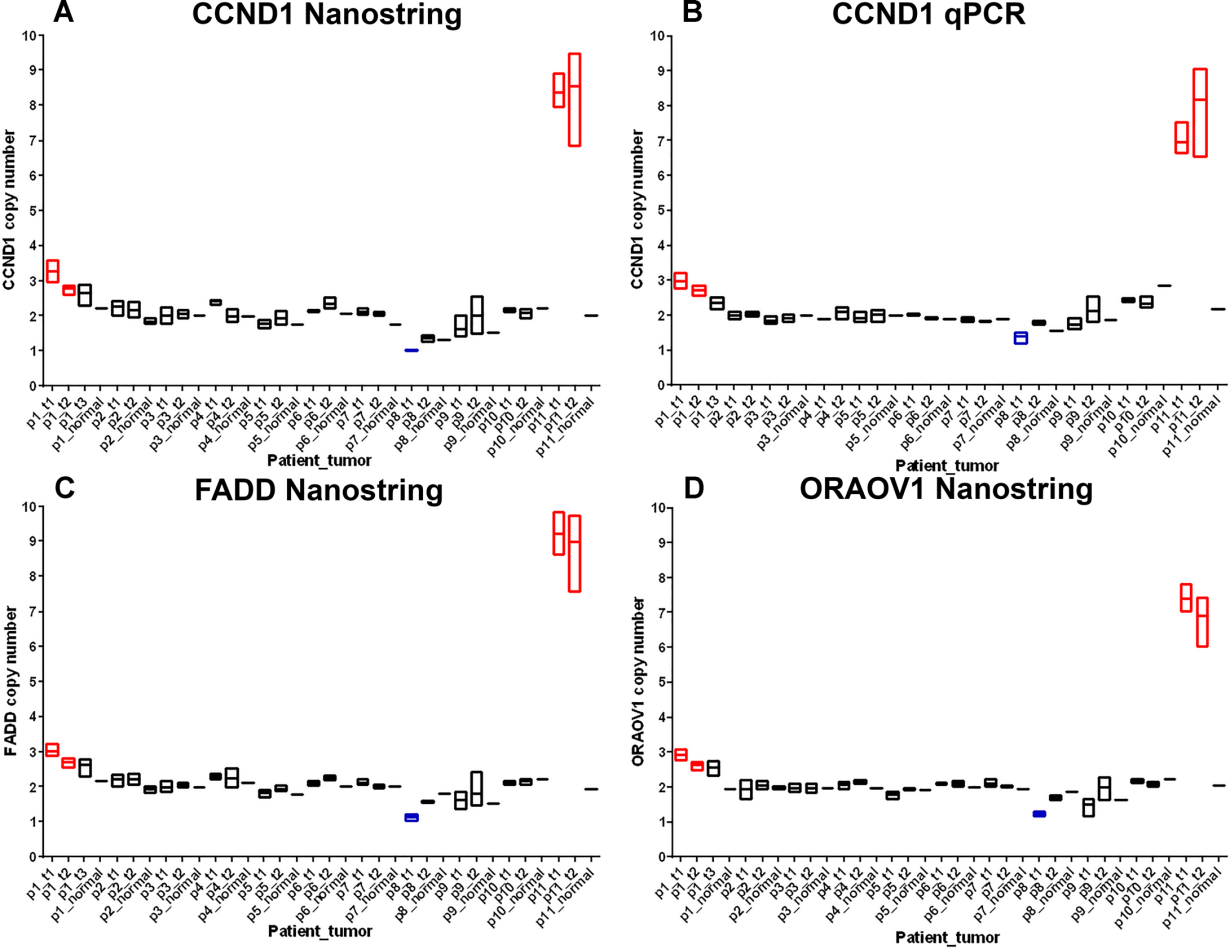
11q13.3 gene copy number in 11 MF ILC patients. Gene copy number was measured in three punches from each focus and a single punch from matched adjacent normal tissue where available. Ends of each box are minimum and maximum copy number and floating bar shows mean copy number. Copy number was measured by Nanostring and qPCR platforms. Patients are labelled p1-11 and foci are labelled t1, t2 and t3 in order of size, hence p1_t1 = patient 1, focus 1. Amplifications are highlighted in red and deletions in blue. A. *CCND1* copy number by Nanostring; B. *CCND1* copy number by qPCR; C. *FADD* copy number by Nanostring; D. *ORAOV1* copy number by Nanostring.

We assessed copy number in all foci in 80 known cancer genes. These data (with the limitation of 80 genes) were suggestive that multiple foci in the same patient are genetically homogeneous. Consistent copy number was observed across all three punches in each primary focus and between primary foci in each patient. We observed relatively few changes in gene copy number across MF ILC, the largest change being amplification of three genes (*CCND1*, *FADD* and *ORAOV1*) mapping to 11q13.3 in two of eleven patients (patients 1 and 11). Copy number data for this region are shown in Fig 2. 11q13.3 amplification has been previously described in breast cancer,[26, 27] specifically ILC, [28–30] and in oral squamous carcinoma where it was reported as prognostic of metastasis [31]. Within our dataset, amplifications were identified in *CCND1*, *FADD* and *ORAOV1* in all three punches from both foci. Patient 11 showed higher copy number, ranging from 6.01 to 9.40 on the Nanostring platform. qPCR at the *CCND1* locus confirmed the amplification in all three punches in both foci, copy number ranging 6.53–9.06. Amplification in patient 1 was more subtle, ranging 2.30–3.60 on the Nanostring platform and 2.17–3.2 with qPCR.

A third patient (patient 8) showed some evidence of deletion at the *CCND1*, *FADD* and *ORAOV1* genes in either one or both foci and consistent low level amplification across all three punches in each focus for *AKT3* mapping to 1q43, copy number ranging 2.68–3.16 (mean 2.88, SD = 0.19) and *MET* mapping to 7q31.2, copy number ranging 2.52–3.93 (mean 3.24, SD = 0.53). In addition, patient 11 showed consistent low level amplification of *ITGB4* at 17q25.1, copy number ranging 3.17–3.66 (mean 3.68, SD = 0.22) and *MYC* at 8q24.21, copy number ranging 2.96–3.51 (mean 3.32, SD = 0.22). All copy number changes are shown graphically across all punches for all 11 patients in S2 Fig.

**Fig 3:**
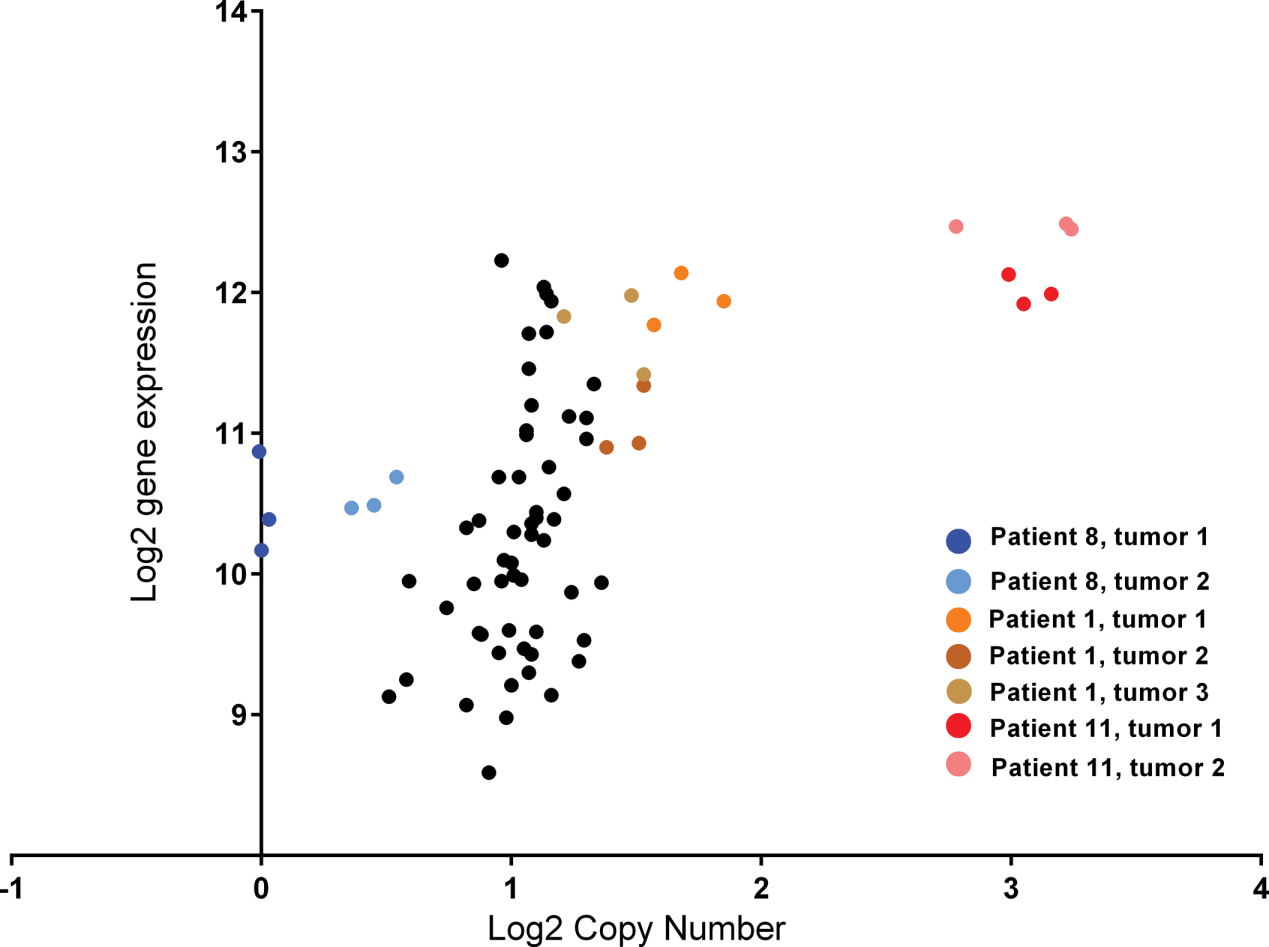
*CCND1* gene expression and gene copy number in 11 MF ILC patients. Patient 8 (blue) deletion; Patient 1 (orange/brown) moderate copy number gain; Patient 11 (red/pink) high copy number gain.

We then proceeded to correlate gene copy number with gene expression at 11q13.3 using *CCND1* which is common to both NanoString PanCancer expression and cancer copy number panels (Fig 3). *CCND1* gene expression was significantly correlated with copy number, Spearman r = 0.57, p<0.0001 when measured across the full sample of eleven patients. When considering just those patients with abnormal copy number: patient 8 (deletion) patient 1 (low level amplification) and patient 11 (high level amplification), correlation improves, Spearman r = 0.88.

#### Differential Pathway Expression in multiple foci within ILC patients

Using two independent methods to measure pathway significance, we did not observe any convincing evidence of differential pathway expression between patient foci across this patient group as a whole, (Fig 1B, S3 Table). However, visual inspection did suggest that the foci in at least 2/11 ILC patients (patients 4 and 7) were heterogeneous at the pathway level for Wnt, PI3K, RAS, Hedgehog, Transcription Misregulation, TGF-Beta, MAPK, STAT and Apoptosis pathways, S3 Fig. This may reflect a biological difference in that the foci in these patients are of different lobular subtypes. In patient 4, both foci are E-cadherin negative, but the larger focus is of the alveolar subtype and the smaller focus is classic lobular subtype. In patient 4, the larger focus is very weakly positive for E-cadherin and of the trabecular subtype and the smaller focus is E-cadherin negative and predominantly of the classic lobular subtype with some mixed trabecular subtype.

Alternatively, for all thirteen pathways in both patient 4 and patient 7, the pathway score of the smaller focus was always more similar to adjacent normal tissue than the larger focus, S4 Fig. These differences could also arise from cellularity in that the smaller focus was of higher normal tissue content (immune, stromal or epithelial). To examine cellularity we plotted copy number for each focus across 80 genes in the Nanostring Cancer CNV panel, S2 Fig. Very few copy number changes were observed in these patients, but where copy number did deviate slightly, the second focus (in both cases the classic lobular subtype focus) was closer to adjacent normal tissue.

### Tumor heterogeneity

Our study design of three punches from every tumor allowed us to examine focal heterogeneity within MF ILC patients and tumor heterogeneity between MF ILC patients, for each gene, with tumor grade (lobular subtype) as a fixed effect. Variance, percentage heterogeneity of all three types (patient, foci, intra-tumor) and p-values for all 730 genes are shown in S4 Table.

There was strong evidence of heterogeneity between-foci within-patients. Of the 730 genes included in the analysis, 466 (64%) had unadjusted heterogeneity (Table 3, pval.F) p-values of <0.05 (with q-values<0.08), and 432 (59%) had a multiple-testing adjusted p-value of <0.05,suggesting that there is within-patient focus-to-focus heterogeneity for over half of the studied genes in addition to any patient-to-patient variability and variability in measures of gene expression among replicate punch samples from the same foci. There was also evidence of heterogeneity from patient to patient in addition to any variability between punches from the same foci and between foci within patients, although to a lesser extent than we observed for multiple foci within patients. Of the 730 genes included in this analysis, 292 (40%) had unadjusted heterogeneity (Table 3, pval.P) p-values of <0.05 (with q-values<0.12). These observations are very relevant when searching for markers of ILC relative to adjacent normal tissue or potential prognostic markers based on gene expression from a single specimen, and may be especially relevant to multifocal disease. This is illustrated in Table 3, where we show a breakdown of heterogeneity measures for *CDH1*, and the other nine genes identified with greater fold changes than *CDH1* in our tumor versus adjacent normal analysis.

**Table 3:** Sources of heterogeneity in MF ILC differentially expressed genes.

Gene	HET.P	HET.F	HET.IT	pval.P	pval.F
*LAMB3*	46.2	41.7	12.1	0.11	1.05E-09
*CDH1*	0.0	73.8	26.2	1.00	3.16E-09
*SPRY2*	0.0	75.1	24.9	1.00	3.42E-09
*NTRK2*	0.0	65.5	34.5	1.00	1.19E-06
*FIGF*	1.5	62.0	36.5	1.00	4.56E-06
*SFRP1*	5.5	54.9	39.6	1.00	4.82E-05
*KIT*	8.6	47.1	44.3	0.86	3.82E-04
*RELN*	15.1	42.4	42.6	0.69	7.53E-04
*MMP7*	19.1	26.1	54.8	0.39	0.04
*PROM1*	54.3	5.5	40.2	0.01	0.44

HET.P, variability between tumors in different patients, expressed as a percentage of total variability from: multiple punches in each tumor, variability between foci within patients and variability between tumors in different patients. pval.P, corresponding p-value for heterogeneity between tumors in different patients. HET.F, variability between foci within patients, expressed as a percentage of total variability. pval.F, corresponding p-value for heterogeneity between foci within patients. HET.IT, variability within tumors (intra-tumor heterogeneity) expressed as a percentage of total variability.

**Fig 4:**
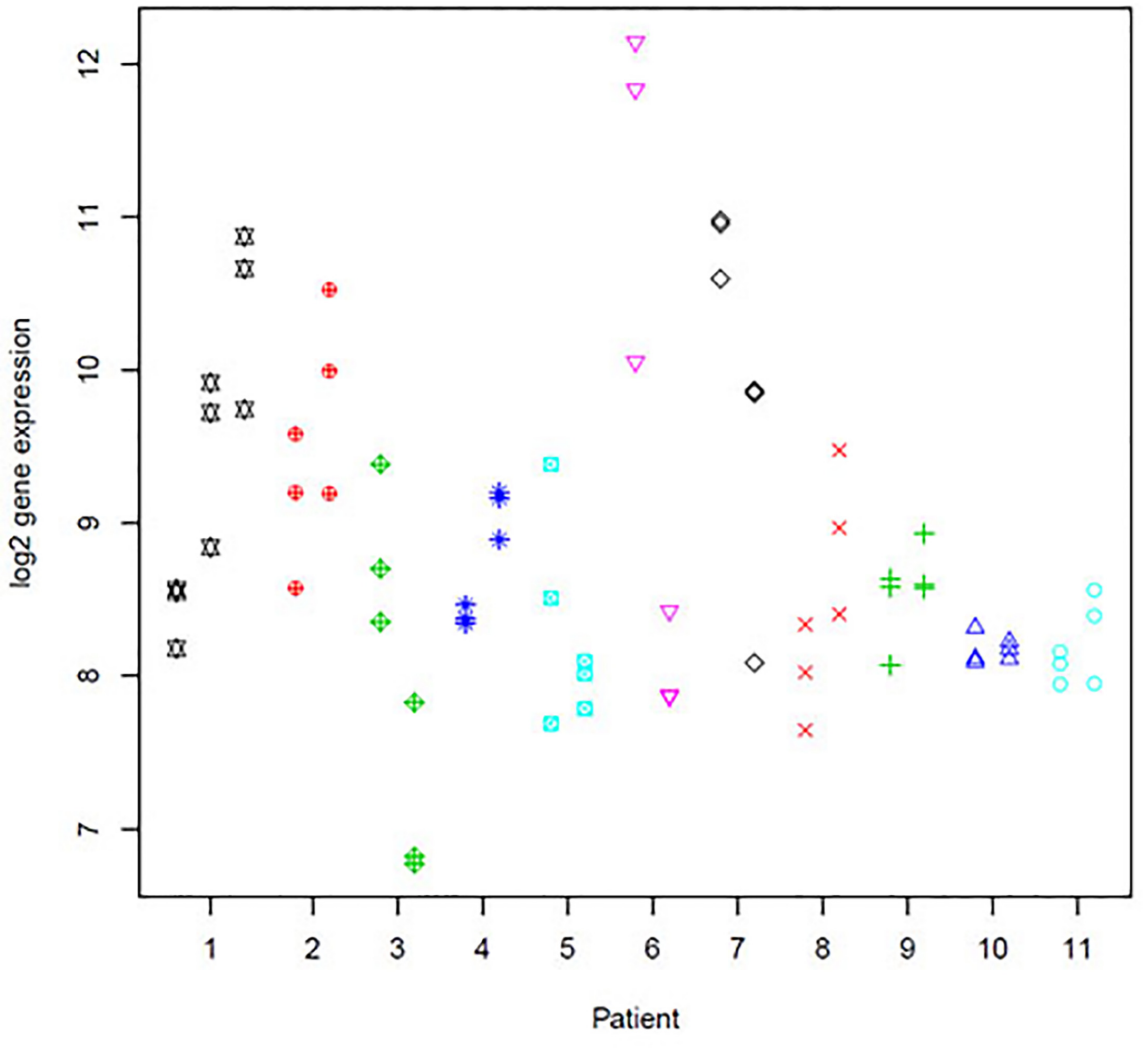
*CDH1* gene expression in 11 ILC patients with multiple foci. Log2 gene expression is plotted for three punches from each foci in each patient. For each patient, foci are shown in order of size with punches from the largest focus always displayed to the left and punches from the smallest focus (for which tissue is available) to the right, in the same order as shown in Table 1.

Gene expression measures for *CDH1* shows very high evidence of within-patient between-tumor heterogeneity (73.8% heterogeneity, p = 3.16x10^-9^). Loss of *CDH1* (E-cadherin) is an important diagnostic feature of ILC. Differential expression analyses of tumor versus adjacent normal tissue confirmed this finding with an absolute fold change of 0.28 in MF foci versus adjacent normal tissue (Table 2). In our heterogeneity analysis of *CDH1* across multiple foci from patients (Fig 4), the median log2 gene expression is 8.56 (compared to 10.65 in adjacent normal tissue), but we see that in 5 of the 11 patients there is no overlap of *CDH1* expression levels between foci, and in patient 6 there are ~3 orders of magnitude difference between foci. This observation is further illustrated in our breakdown of heterogeneity in Table 3 showing the greatest source of heterogeneity for this gene is between foci within patients (73.8%). This trend is also reflected for the nine other genes that were significantly down-regulated relative to adjacent normal tissue, with larger fold change than observed for *CDH1*.

## Discussion

The molecular/genomic aspects of multifocal (MF) invasive lobular carcinoma (ILC) are poorly understood, despite the fact that these patients have significantly worse outcome than ILC patients with unifocal disease [11–14]. In this study we used detailed molecular characterization of multiple foci in a set of 11 MF ILC patients, all of whom were ER+ and HER2- in both foci.

Our first analyses of differential gene expression of tumor versus adjacent normal tissue were generally consistent with published studies of ILC [32–35]. We identified 253/730 genes from canonical cancer pathways that were significantly different, with estimated FDR = 14%. Loss of E-Cadherin (*CDH1*) is associated specifically with ILC and is an important diagnostic feature [32]. In this dataset of 11 MF ILC patients, *CDH1* was significantly down-regulated, p = 4.6x10^-4^, q = 0.0093, absolute fold change 0.28.

Due to small sample size and different platforms used, the overlap of biomarkers (other than *CDH1*) between published studies of ILC genes is small. A meta-analysis of five gene expression studies identified *THBS4* as a potential ILC biomarker.[34] *THBS4* gene expression was also up-regulated in our ILC patients relative to adjacent normal tissue (absolute fold change 2.86, p = 0.027, q = 0.10). Korkola et al [33] defined 11 genes as capable of differentiating ILCs from ductal carcinoma, of which three (*CDH1*, *SPRY1*, *THBS4*) are present in the PanCancer pathways panel. All three genes were significantly differentially expressed relative to adjacent normal tissue in our sample set, p-values 4.6x10^-4^, 0.018 and 0.027 respectively, with estimated FDR associated with p<0.027 of 10%, (although we note that *SPRY1* expression was lower in ILC relative to adjacent normal in our study and higher in ILC relative to IDC in the Korkola study). Turashvili et al [35] identified a number of genes as being significantly differentially expressed between lobular and ductal carcinoma relative to normal epithelial tissue, of which one, *COL3A1* is also present on the PanCancer pathway panel. In agreement with this study, *COL3A1* is significantly up-regulated in our ILC patients with a fold change of 4.5 and p = 5.76x10^-5^ that remained significant even after adjustment for multiple testing.

The PanCancer pathway panel includes multiple collagen genes and our data also showed significantly higher expression of *COL11A1*, fold change 24.99, p = 0.001, *COL5A1*, fold change 3.91, p = 2.0x10^-8^ and *COL1A1*, fold change 8.40, p = 5.65x10^-8^ in ILC tumor relative to adjacent normal tissue. Enhanced expression and deposition of collagens are associated with tumor development, progression [36–38] and specifically, breast cancer invasion and aggressiveness [39]. Collagens are the main structural extracellular matrix proteins and perhaps upregulation of these genes in multifocal ILC is related to the upregulation of *THBS4*, an extraceullalar glycoprotein. *THBS4* plays an important role in interactions with the extracellular matrix and has also been shown to be expressed at higher levels in cancer associated stroma relative to normal stroma, with highest expression in tumors rich in stromal content, (ILC, ER positive low grade IDC; luminal A and normal-like subtypes) [34]. These findings suggest that increased *THBS4* expression in breast cancer-associated extracellular matrix contributes to the activated stromal response exhibited during tumor progression and that this may facilitate invasion of tumor cells. Our pathway analysis of ILC tumor versus adjacent normal is supportive of these findings in that the collagen genes and *THBS4* map to the PI3K pathway (the most dysregulated pathway in our tumor versus normal dataset). PI3K genes are expressed in both tumor and stromal cell types, allowing cross-talk between these cell types to modify the surrounding tumor microenvironment and promote tumorigenesis [40].

Our study identified *FIGF*, *RELN*, *PROM1*, *SFRP1*, *MMP7*, *NTRK2*, *LAMB3*, *SPRY2 and KIT* as being both significantly down-regulated in MF ILC (absolute fold changes 0.06, 0.12, 0.13, 0.15, 0.17, 0.22, 0.23, 0.24, 0.26, respectively) similar to *CDH1* (absolute fold change 0.28) and significant with q<0.05. Given our study design of ILC tumor versus normal, rather than ILC versus IDC, it is possible that these genes are not specifically markers of ILC. We note that Turashvili *et al* [35] reported expression of *SFRP1* to be significantly down-regulated in both ILC and IDC relative to matched normal lobular and ductal tissue respectively, and expression of *MMP7* to be down-regulated in ductal carcinoma only. However, we also note that fold changes for all nine of these genes are in the same direction and of similar magnitude to *CDH1*, a hallmark of ILC. One possibility is that these decreases in expression are related to loss of *CDH1* and are potential therapeutic targets in ILC. Four of these genes, (*FIGF*, *RELN*, *LAMB3* and *KIT*), map to the PI3K pathway, two of which (*FIGF* and *KIT*) map to both PIK3 and RAS pathways. *NTRK2* (Tyrosine kinase B neurotrophin receptor) functions in the MAP kinase pathway. Its kinase activity reportedly contributes to disease progression by inhibiting anoikis and promoting epithelial to mesenchymal transition, and PIK3 is an important downstream target of this cell survival pathway [41, 42]. *SPRY2*, an inhibitor of RAS/mitogen signaling, has been previously associated with prognosis of breast cancer [43]. We also examined differential pathway expression at the level of MF ILC versus adjacent normal tissue. All pathways were significant at p<0.05. We observed a high correlation between two independent measures of pathway significance allowing pathways to be ranked in order of significance. The most significant pathways were PI3K and cell cycle, although DNA repair, TGFβ, RAS, Wnt and MAP kinase were also highly significant, with PI3K and RAS showing the largest fold change. These data are also in agreement with current thinking that E-Cadherin-mediated adhesion inhibits tyrosine kinase receptor signaling; whereas loss of E-Cadherin, a salient feature of ILC, results in activation of receptor tyrosine kinase signaling pathways [44, 45].

We next sought to systematically examine differences between multiple foci within ILC patients. Firstly, we examined gene copy number across 80 known cancer genes. Copy number analysis showed a high level of consistency from multiple punches within each focus and between foci in the same patient. Two of eleven patients showed gain in copy number at 11q13.3. Amplification of three genes at this locus (*CCND1*, *FADD* and *ORAOV1*) was identified consistently in both foci of one patient (~8 copies), with a second patient showing small gain (3 copies) in both foci. Expression of *CCND1* was also observed to be significantly up-regulated at the level of mRNA (q = 0.008) relative to adjacent normal tissue. *CCND1* gain has been previously observed in both ILC and IDC [26, 28–30] with increased frequency in ILC relative to IDC [29]. The observation of this amplification consistently in both foci (from multiple sampling of three different punches in each foci), and the lack of other chromosomal aberrations is in line with previous studies of multifocal breast cancer, suggesting that the majority of multifocal lesions are clonally related [22–25]. We also observed some evidence for deletion of the same region in a single patient in either one or both foci, although given the difficulty in reliably estimating small copy number changes (particularly in samples from FFPE material), this could be due to artifact, and to our knowledge, deletion of *CCND1* has not previously been reported.

Our copy number data suggest that multifocal ILC foci are, for the most part, very similar with respect to these genomic features. However, our unique study design of genomic data from three punches from each tumor in each patient allowed us to specifically separate intra and inter-tumor heterogeneity within patients and tumor heterogeneity between patients.

This analysis revealed strong evidence of within patient focus to focus heterogeneity for greater than half of the studied genes in addition to any between patient variability and variability in gene expression measures among punches from the same tumors after adjustment for tumor size. Put into the context of *CDH1*, a known hallmark of ILC, we observed significant loss of *CDH1* expression across our sample of 11 MF ILC patients relative to adjacent normal tissue. We also observed, significant heterogeneity (73.8%, q = 9.60x10^-8^) of *CDH1* gene expression between foci within the same patient, with one patient (patient 6) showing lower *CDH1* expression by three orders of magnitude in the larger focus. Both foci in this patient were of the classic lobular subtype, and within the same quadrant (3cm apart). It is possible that this observation is an artifact due to different amounts of contaminating normal cells. However, our gene expression analyses showed relatively low level intra-tumor heterogeneity and our copy number analysis was uninformative: we did not observe any copy number changes <1.5 or ≥3.0 for this patient in either focus and our copy number panel of 80 genes did not include *CDH1* or any other genes on chromosome 16.

Of the nine genes that were significantly down-regulated in tumor relative to adjacent normal at greater magnitude than *CDH1*, we also observed significant levels of within patient focus to focus heterogeneity for eight of nine genes. The same genes did not show significant heterogeneity between patients, which is likely why they we were able to detect significant difference in our tumor versus adjacent normal analysis. This opens the question, how many potential disease markers are missed due to patient heterogeneity in study designs of tumor versus normal, especially when using single specimens per tumor. Our study gives some estimation of this when ranking genes by the percentage of patient heterogeneity (S4 Table), which results in 13 genes: *PPP2R2C*, *RAC1*, *IL20RB*, *MAPT*, *PPP2CB*, *MGMT*, *DDIT4*, *MAPK3*, *ZBTB16*, *FGFR3*, *LAMA1*, *RNF43* and *IL23R*, with >80% heterogeneity between patient tumors, and very little heterogeneity within tumors or between foci within the same patient.

Current literature suggests loss of *CDH1* and other genes that potentially differentiate between ILC and IDC, promote epithelial to mesenchymal transition and activation of receptor tyrosine signaling pathways. Our data demonstrate an additional level of complexity of within patient foci heterogeneity and between patient tumors that could mask potential prognostic factors of MF ILC.

The main limitations of these observations are the limited sample size (eleven patients), and lack of point mutation analysis, as heterogeneity of driver mutations between foci would suggest a source of metastatic potential in MF ILC, that we were unable to address by gene expression analyses. Despite these limitations, this is the most detailed molecular study to date of MF ILC patients, all of whom were ER+ and HER2-. The importance of sequencing analyses of tumor heterogeneity and evolution was recently highlighted in two studies which included patients with MF ductal carcinoma (but not MF ILC). Desmedt et al [46] observed genomic heterogeneity between foci in 12/36 patients with MF IDC, despite similar pathological features. Yates et al [47] examined two to five foci from each of four patients with MF IDC, observing mutations in known driver genes that were private to one focus in three of four patients. They also found many private mutations with high variant allele fractions within individual foci, suggesting the occurrence of complete ‘clonal sweeps’ that replaced all other tumor cells within the focus. Our findings of genomic heterogeneity in MF ILC via gene expression analyses, multiregion sequencing studies of MF IDC [46, 47] and key questions established from an International meeting on the extent of tumor heterogeneity [48] suggest further exploitation of tumor heterogeneity in MF breast cancer will be crucial to our ability to design and select effective therapies and curtail treatment resistance.

## Supporting Information

S1 Fig Patient 7, tumor 1: A) Hematoxylin and Eosin staining, trabecular subtype. B) E-cadherin staining, weakly positive. C) Cytoplasmic p120 staining. Patient 7, tumor 2: D) Hematoxylin and Eosin staining, dominant classic lobular with some mixed trabecular. E) E-cadherin staining, negative. F) Cytoplasmic p120 staining.
(TIF)

S2 Fig Copy number plots by chromosome for each patient. (PDF)

S3 Fig Pathway dysregulation scores for 11 MF ILC patients. Boxes represent maximum, minimum and mean score observed from 3x1.5mm core punches from each tumor and a single punch for adjacent normal tissue where available. Patients are labelled p1-p11 and tumors are labelled T1 and T2. Patient 1 has three tumors labelled p1_T1, p1_T2 and p1_T3. Patients 4 and 7 with the most differences in pathway score between T1 and T2 are highlighted in red and blue respectively.
(PDF)

S4 Fig Pathway dysregulation scores for patient 4 and patient 7. Each score from 3 core punches from each tumor and a single punch for adjacent normal tissue. Bars represent maximum, minimum and median score.
(TIF)

S1 Table Differential gene expression analysis, tumor versus adjacent normal for 730 genes in the Nanostring PanCancer panel. (XLSX)

S2 Table Pathway significance analysis of thirteen cancer pathways in tumor versus adjacent normal tissue. (DOCX)

S3 Table Pathway significance analysis of thirteen cancer pathways between foci. (DOCX)

S4 Table Tumor heterogeneity analysis. This table shows the results of the heterogeneity linear mixed model analysis. The first column shows the gene name; columns B-D show the estimated standard deviations of the random effects for patient (σ_P_), tumor foci (σ_F_), intra-tumor punch sample (σ_IT_) respectively. Columns E-G shows the estimated percent heterogeneity corresponding to each of these as described in the methods. Column H shows the p-value from the likelihood ratio test of the null hypothesis of no between patient variation, H_0_: σ_P_ = 0. Column I shows p-values adjusted for multiple testing using the Holm method, and column J shows q-values that are estimates of false discovery rate at each p-value cut-off. Columns K to M similarly show p-values and q-values corresponding to the test of the null hypothesis of no within-patient, between foci heterogeneity: H_0_: σ_F_ = 0.
(XLSX)
