# Supplementary figures and images for: Assessment of Tumor Heterogeneity, as Evidenced by Gene Expression Profiles, Pathway Activation, and Gene Copy Number, in Patients with Multifocal Invasive Lobular Breast Tumors
Source: PLoS One. 2016 Apr 14;11(4):e0153411. doi: 10.1371/journal.pone.0153411 (PMC4831790; doi:10.1371/journal.pone.0153411)

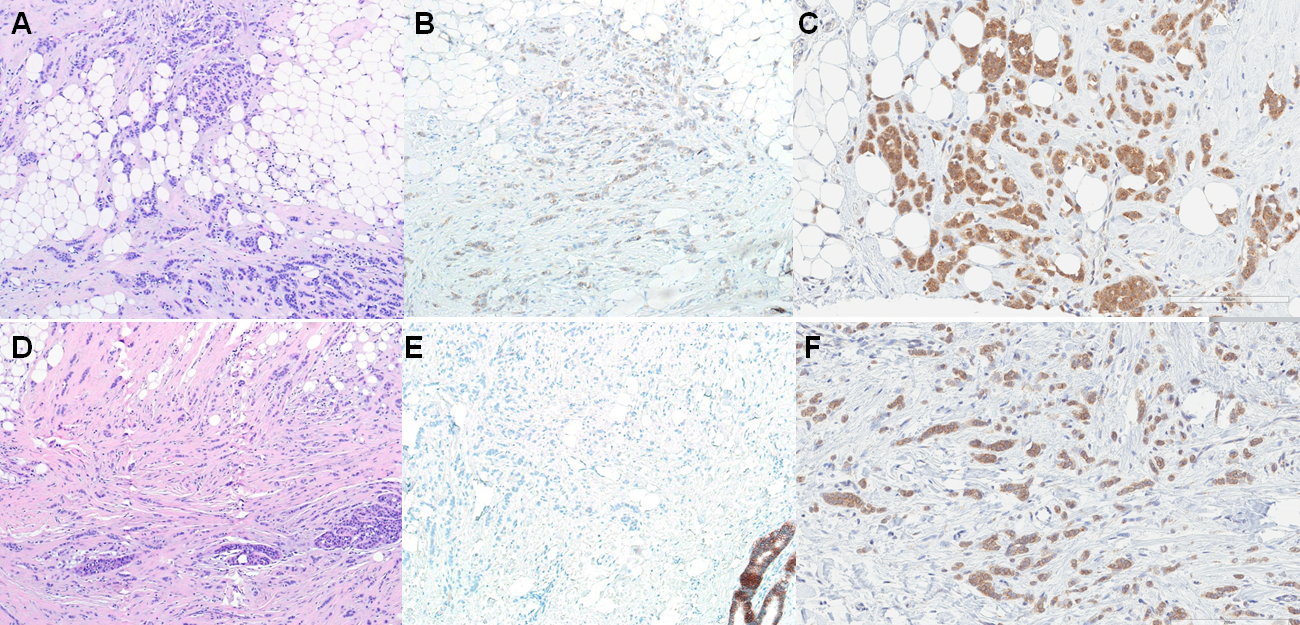

Supplement: S1 Fig — Patient 7, tumor 1: A) Hematoxylin and Eosin staining, trabecular subtype. B) E-cadherin staining, weakly positive. C) Cytoplasmic p120 staining. Patient 7, tumor 2: D) Hematoxylin and Eosin staining, dominant classic lobular with some mixed trabecular. E) E-cadherin staining, negative. F) Cytoplasmic p120 staining. (TIF) [file pone.0153411.s001.tif]

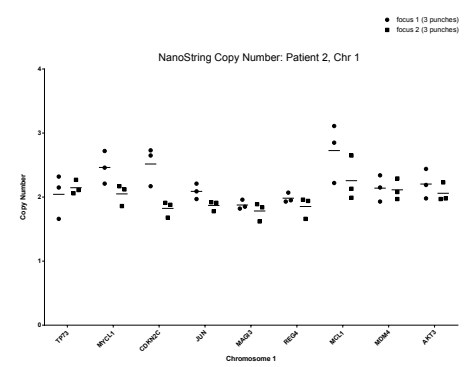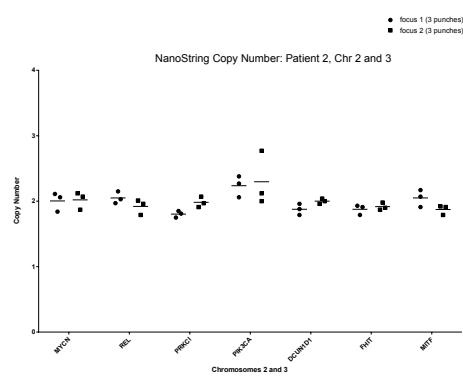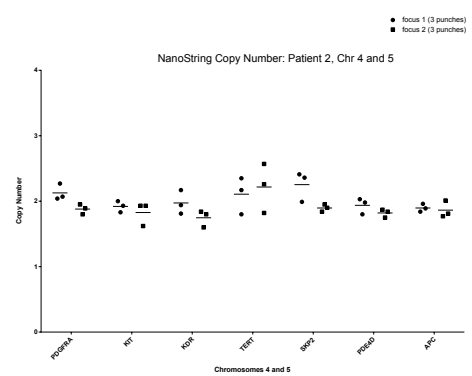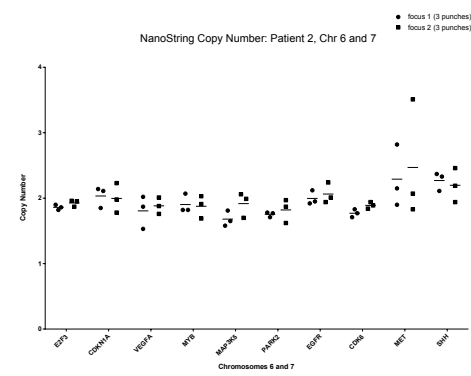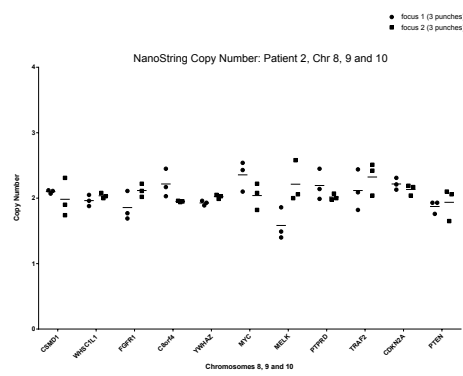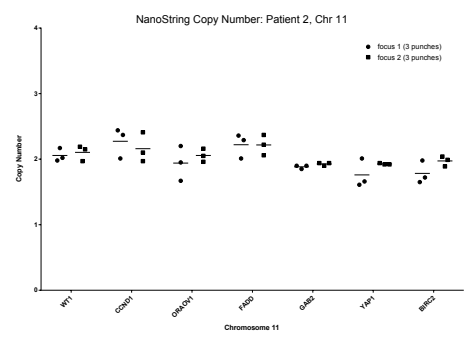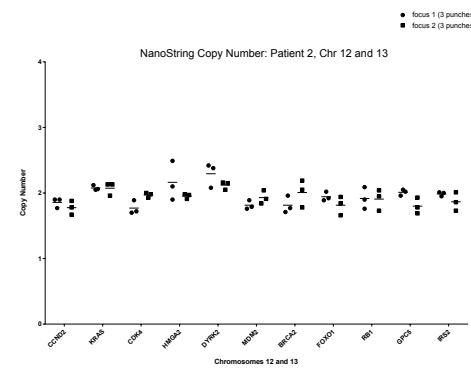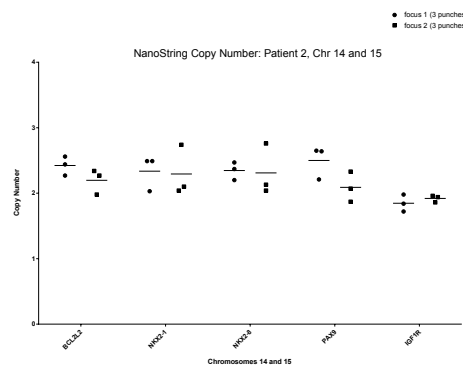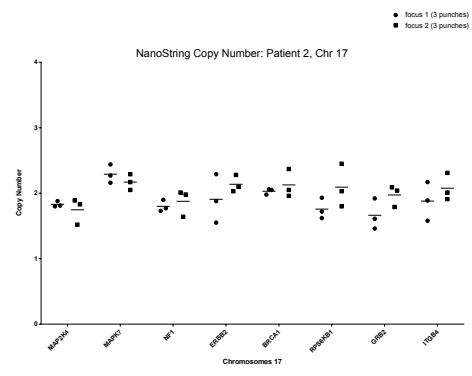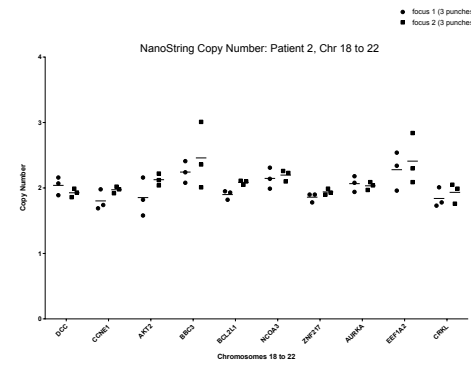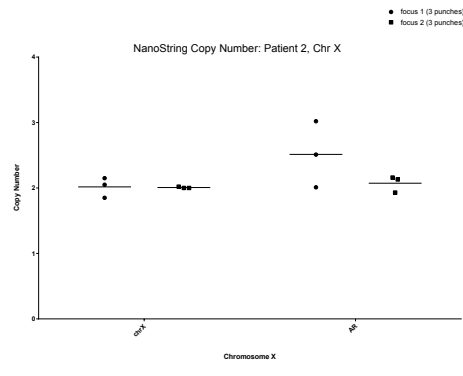

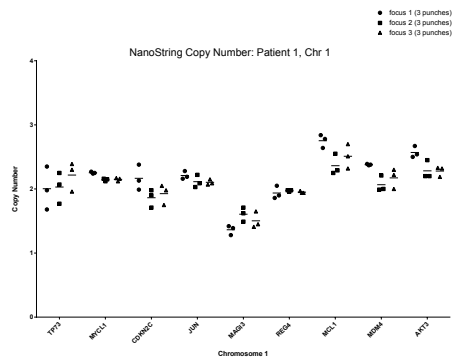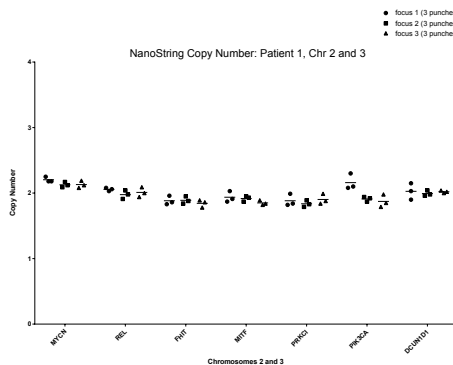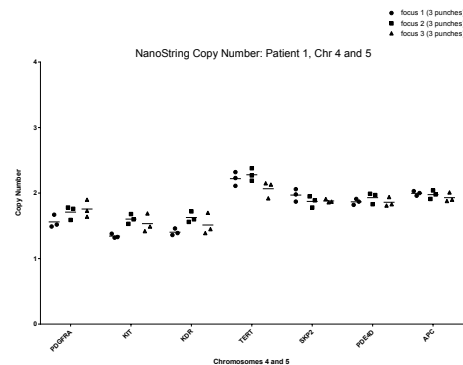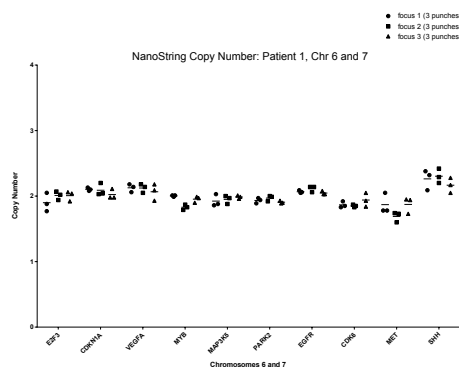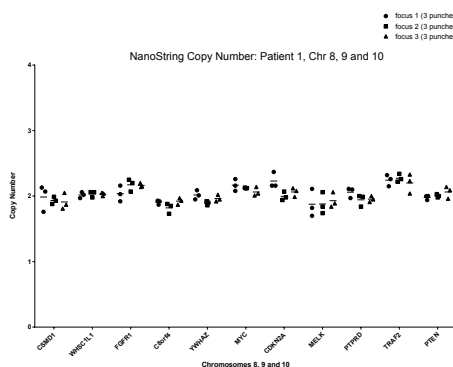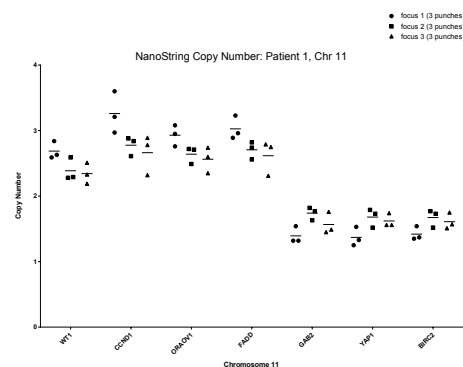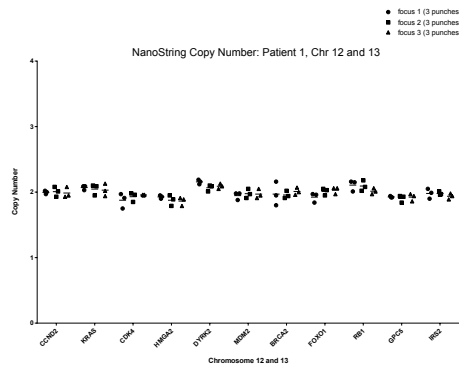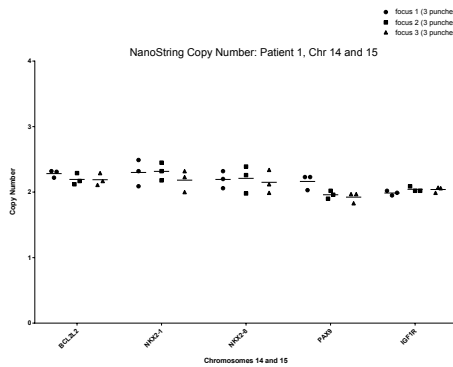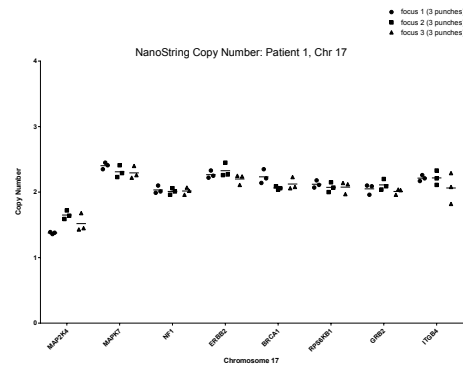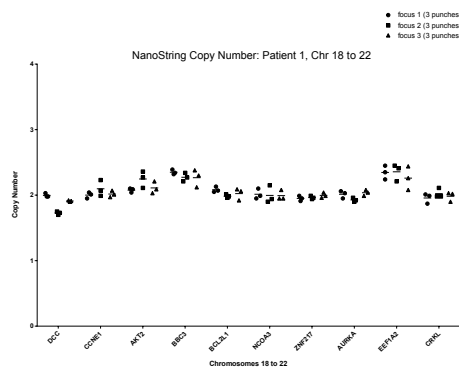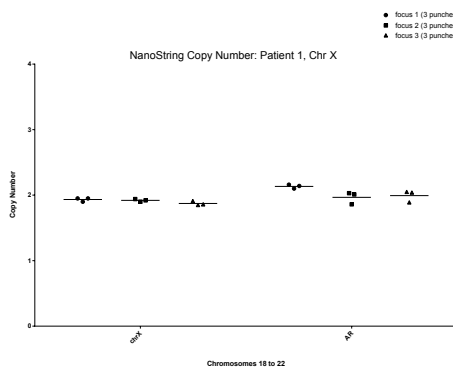

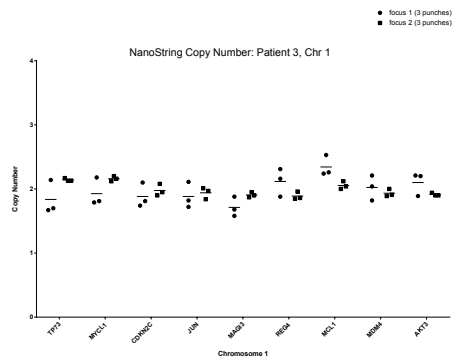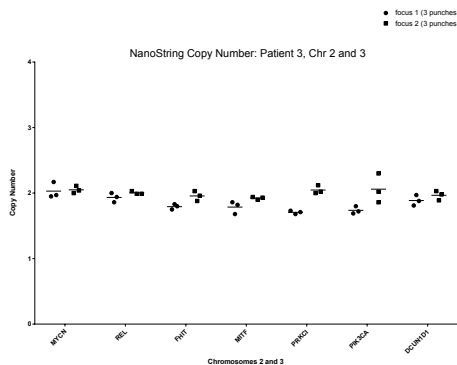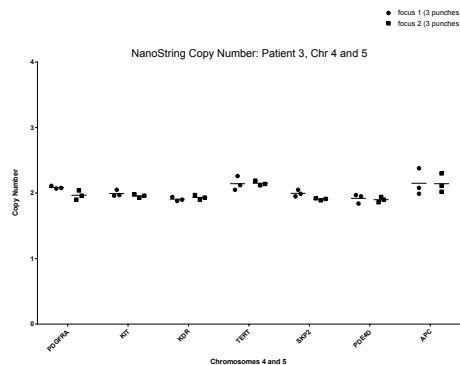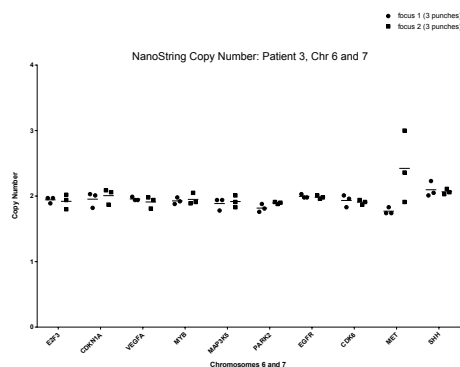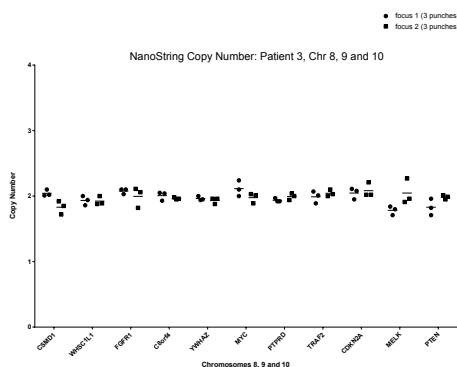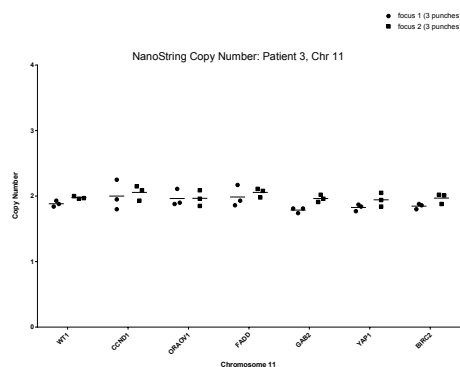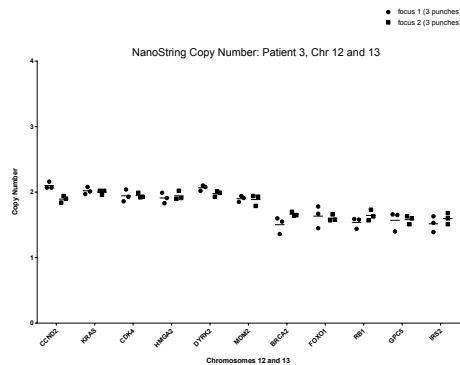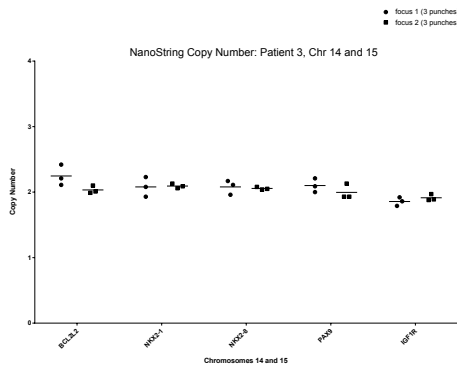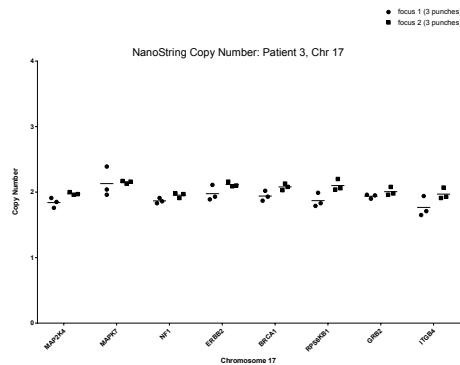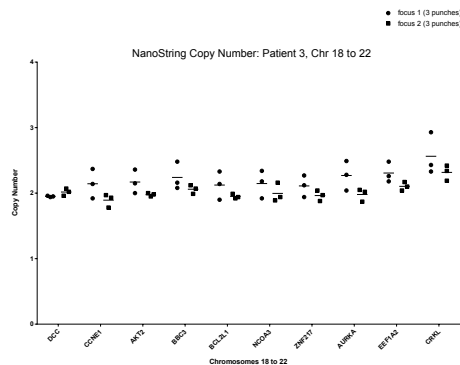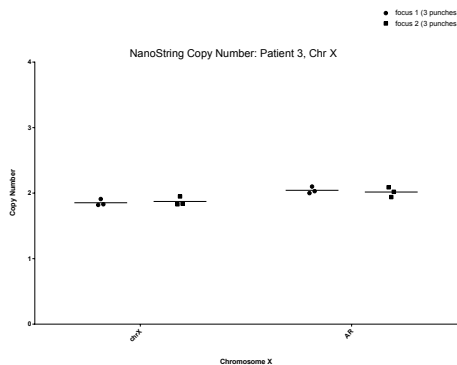

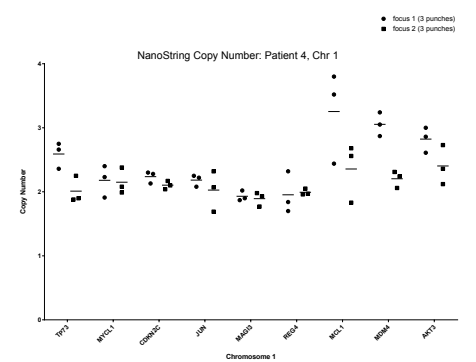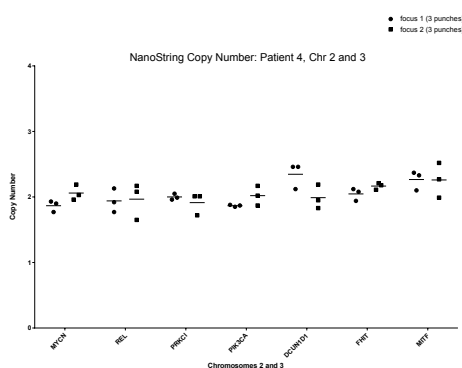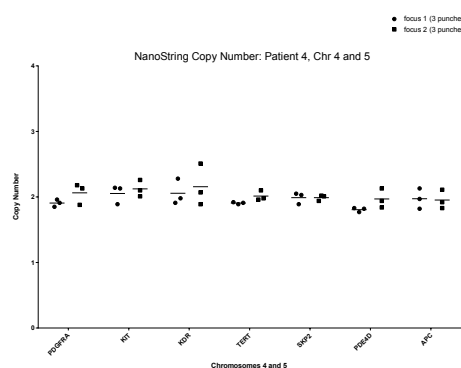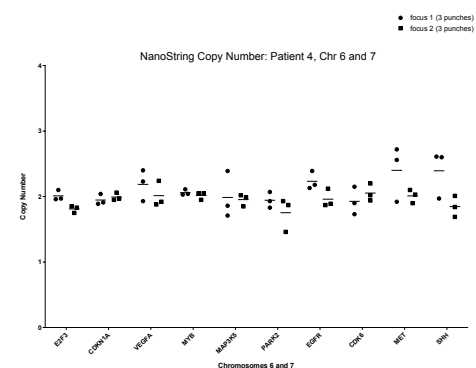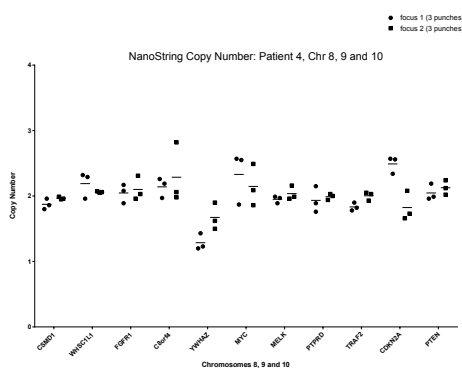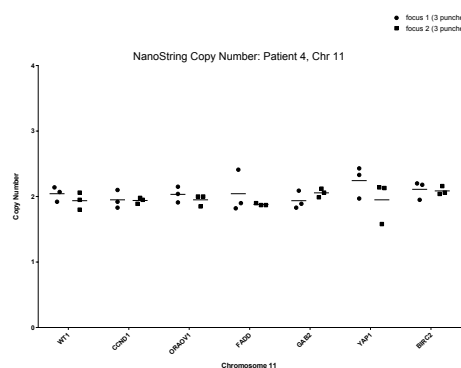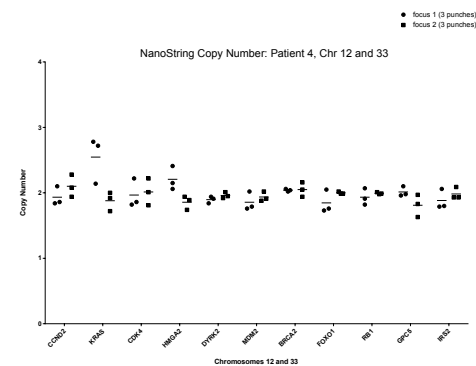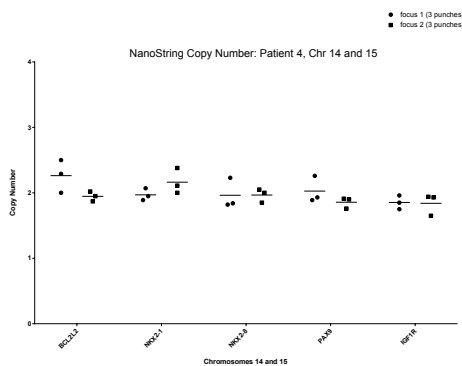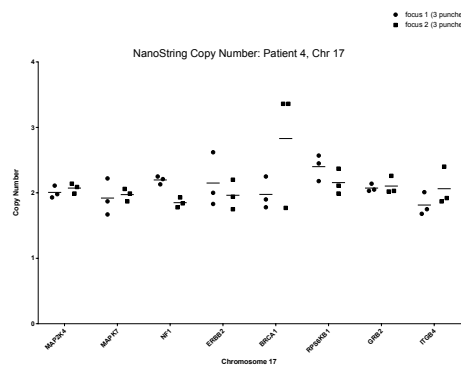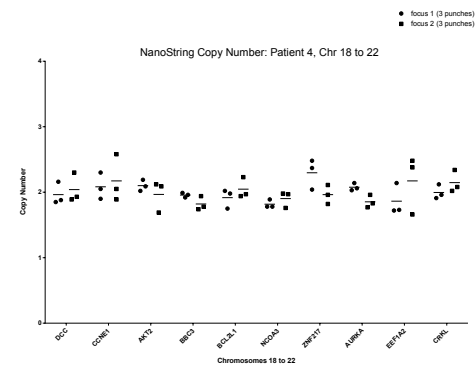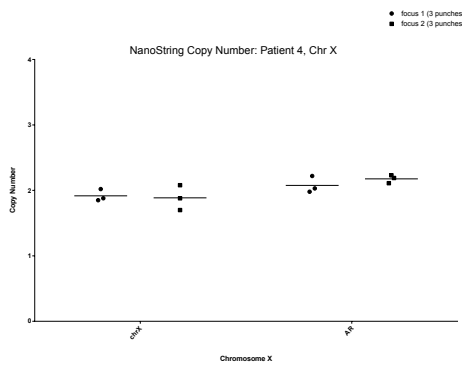

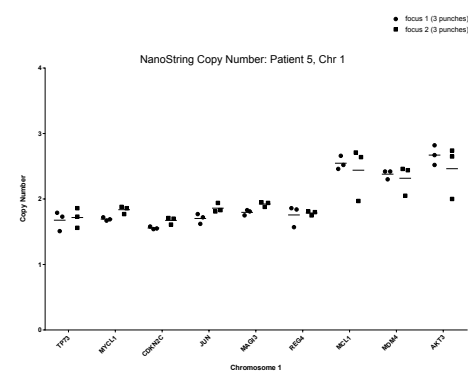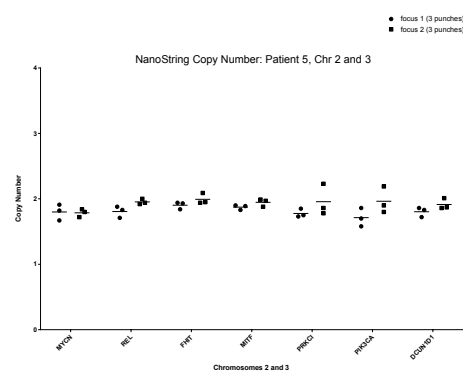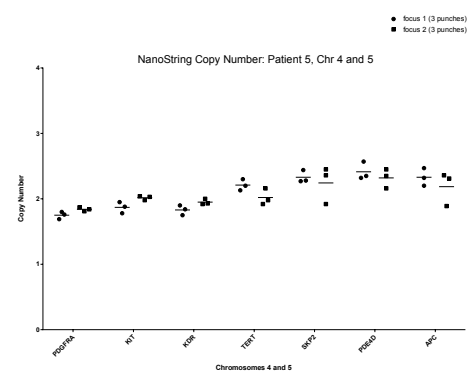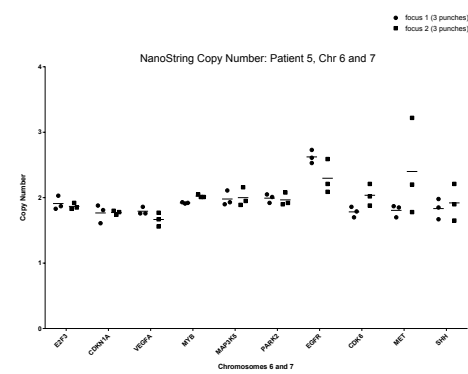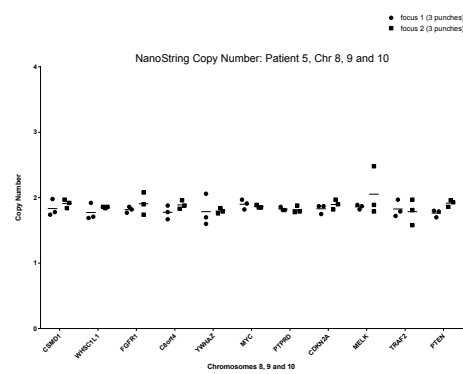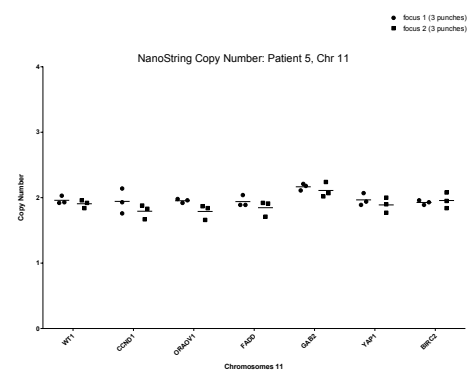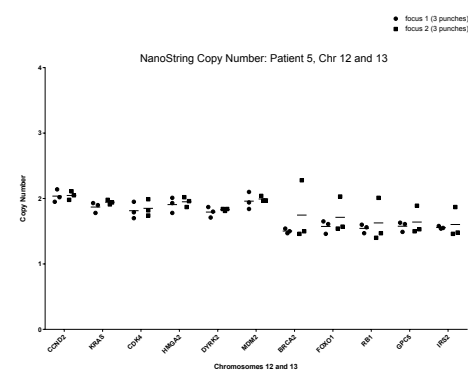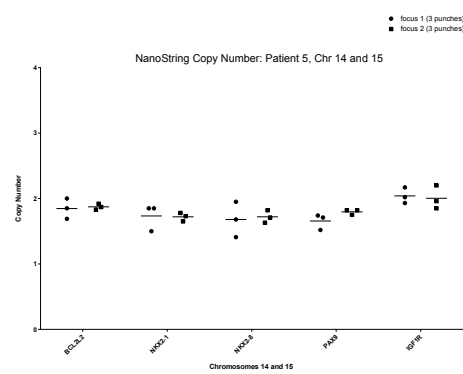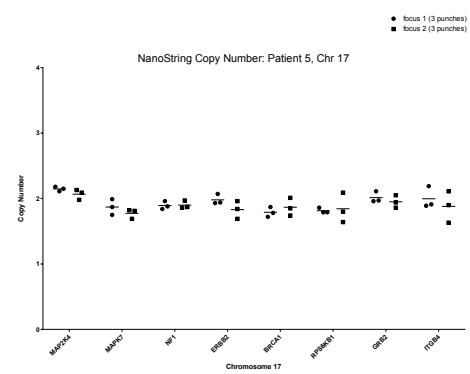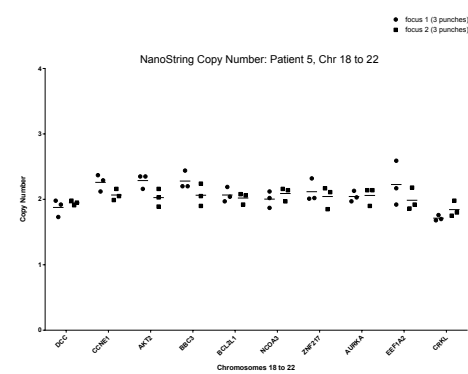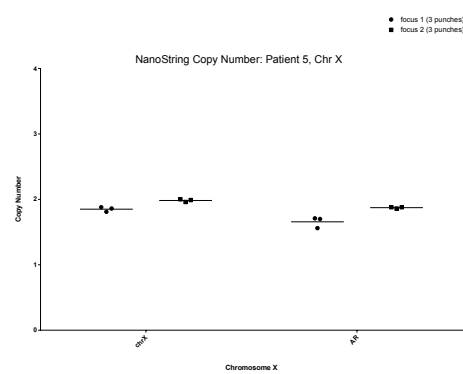

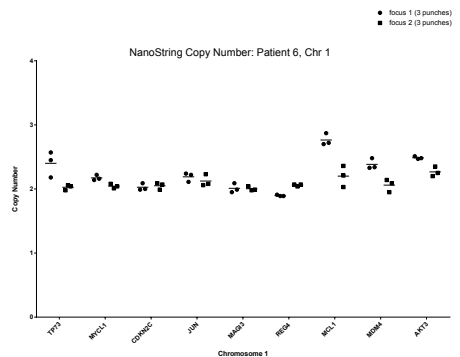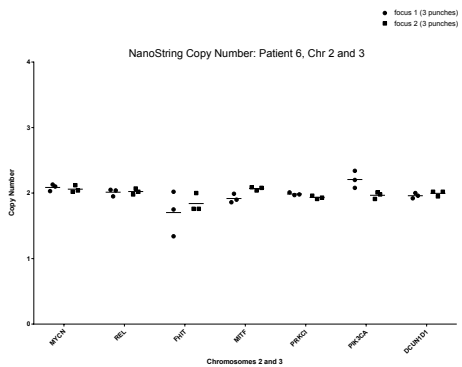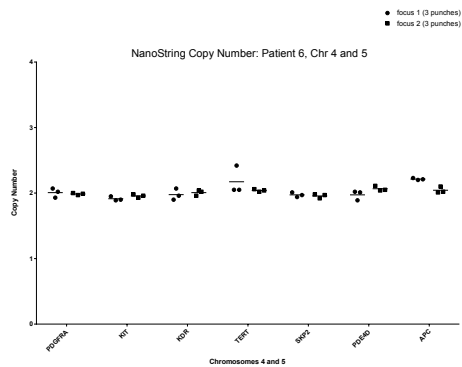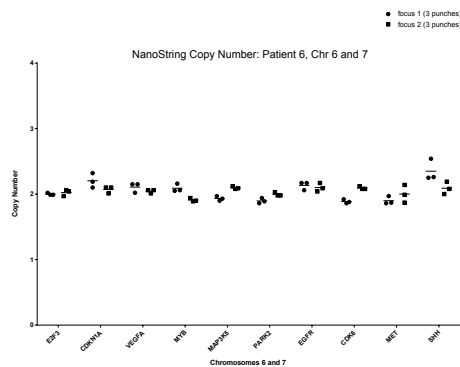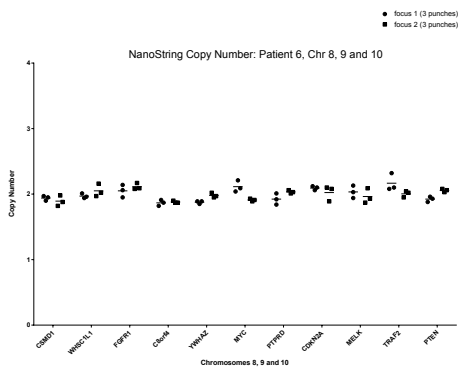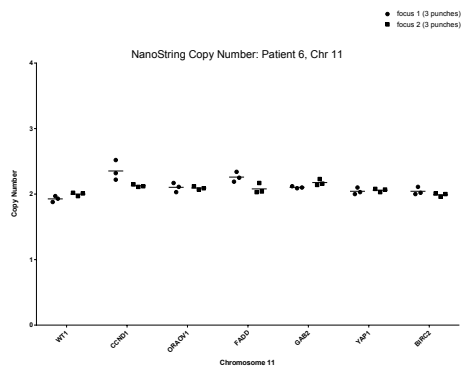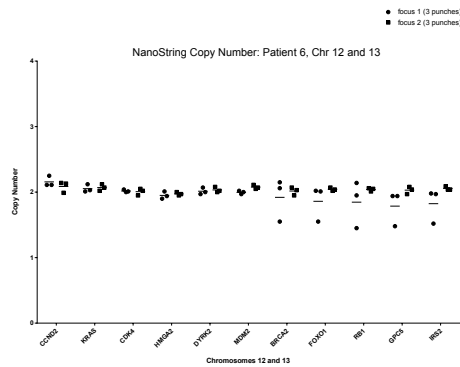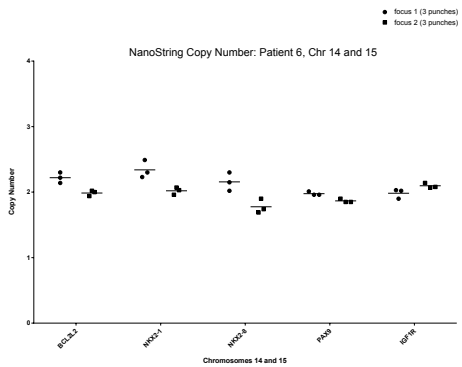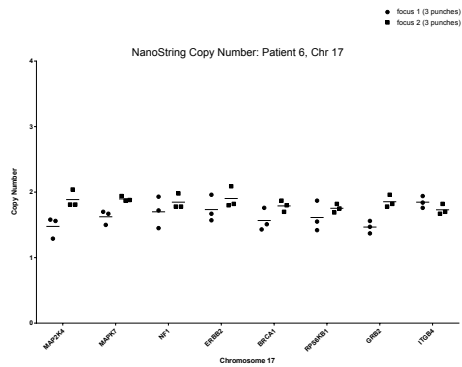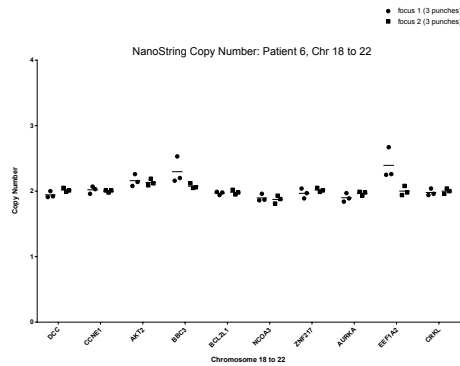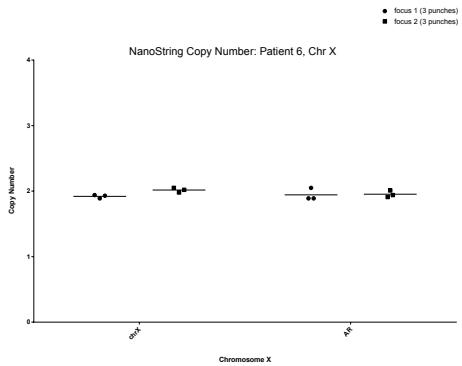

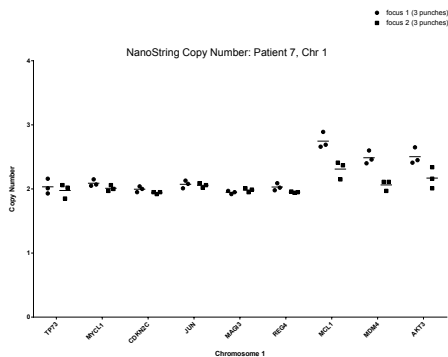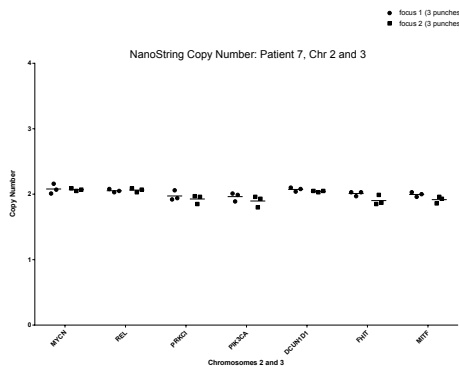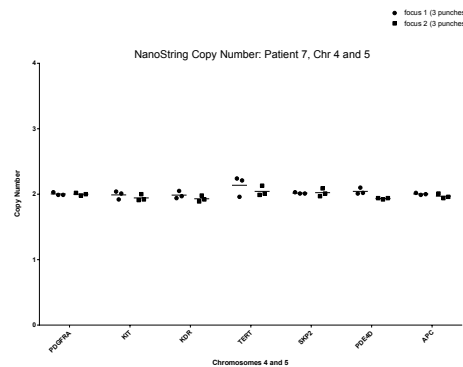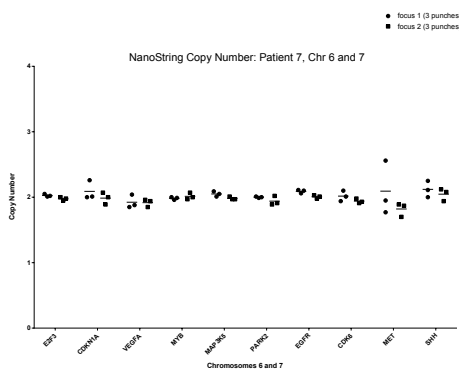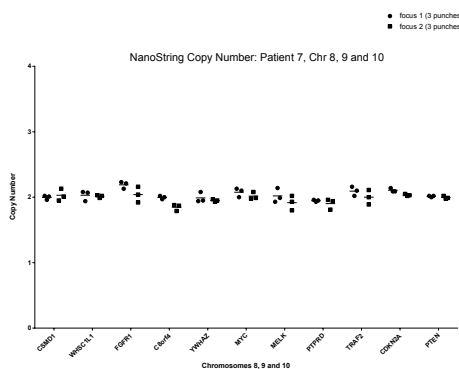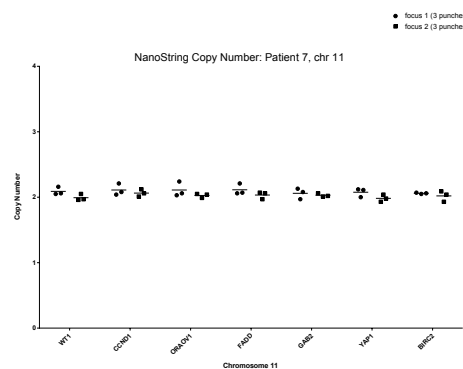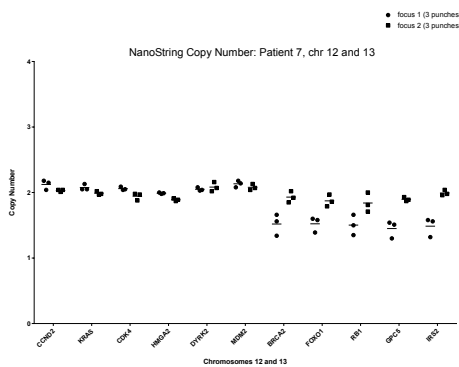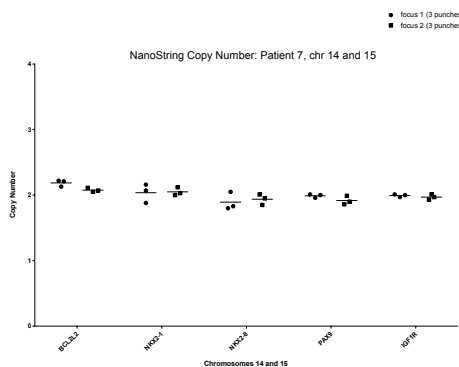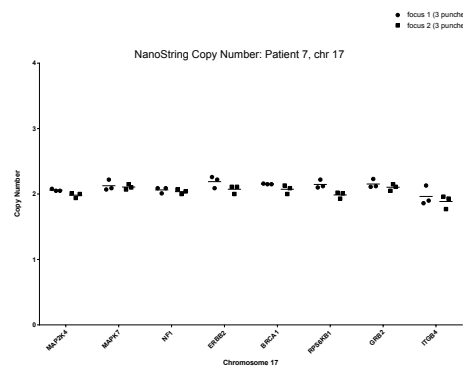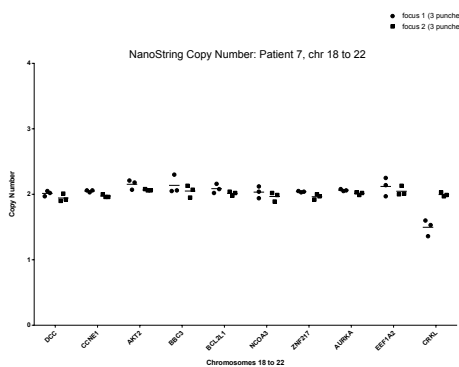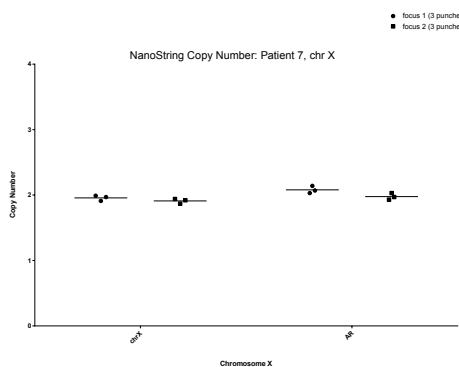

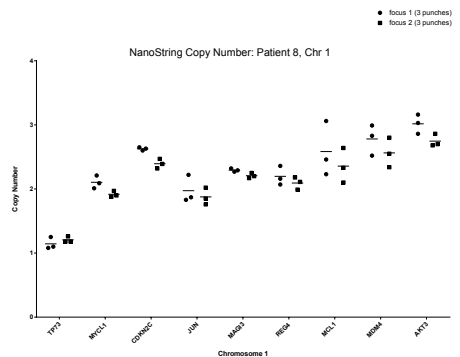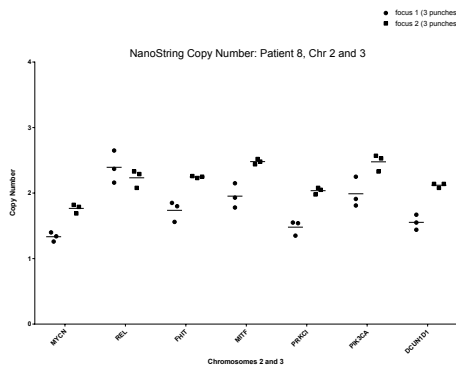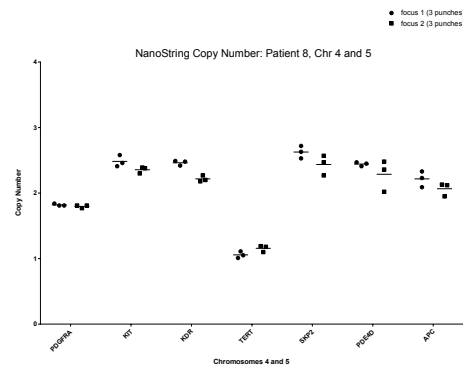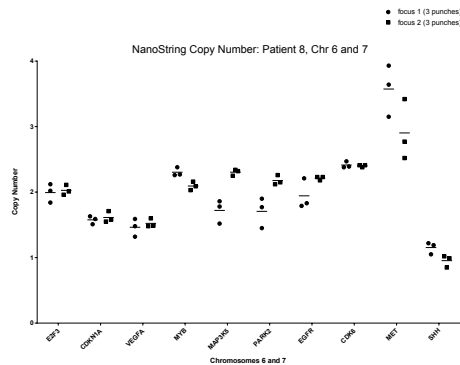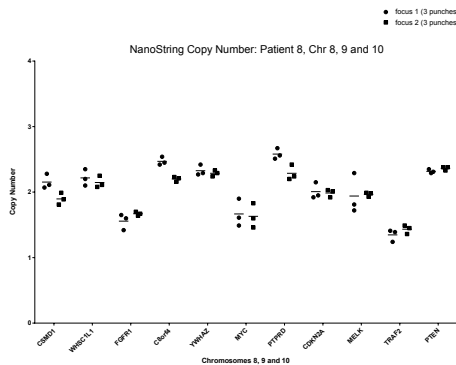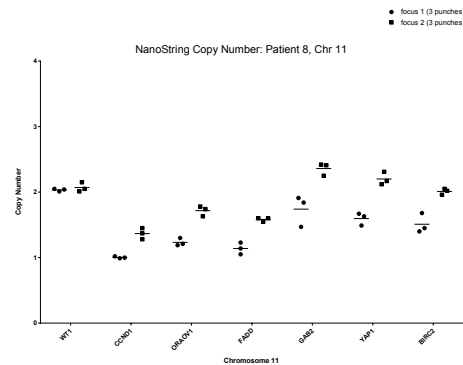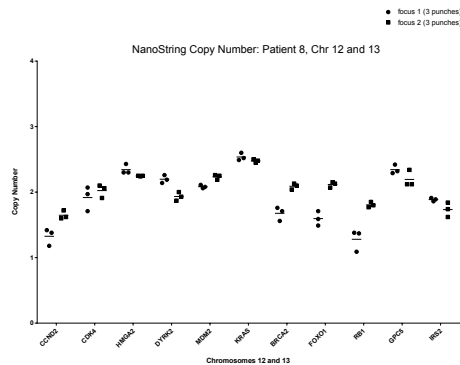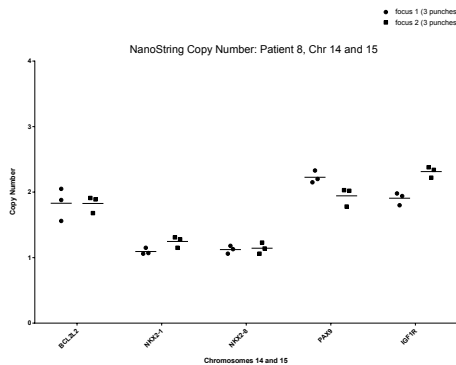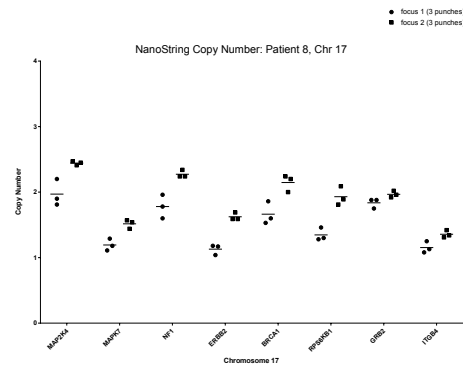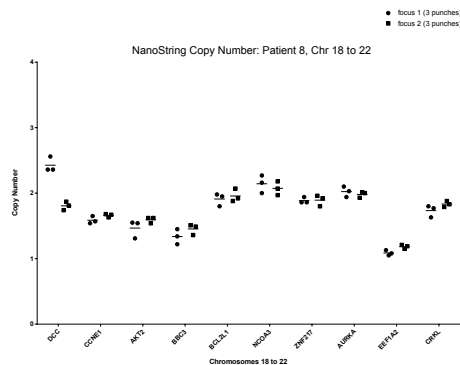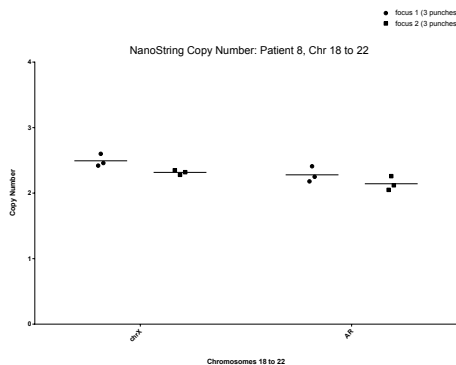

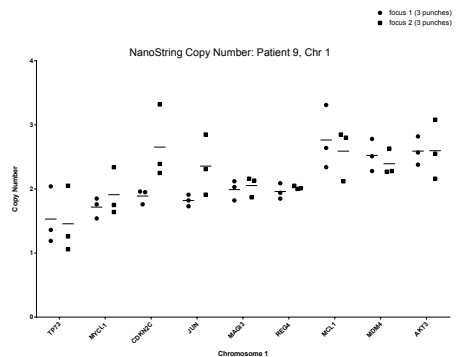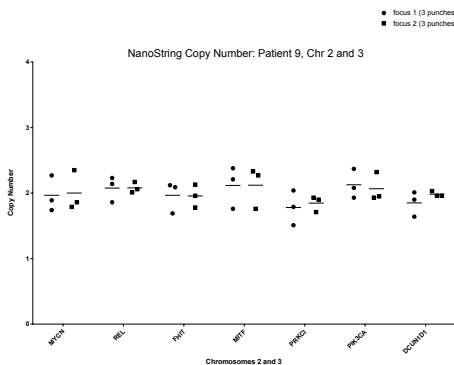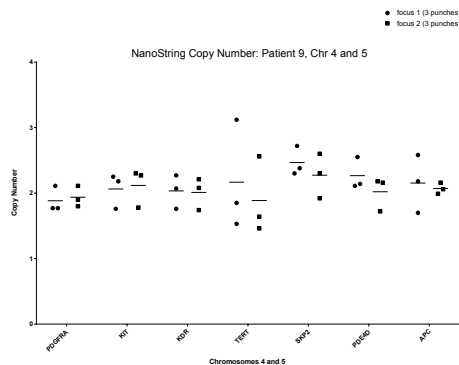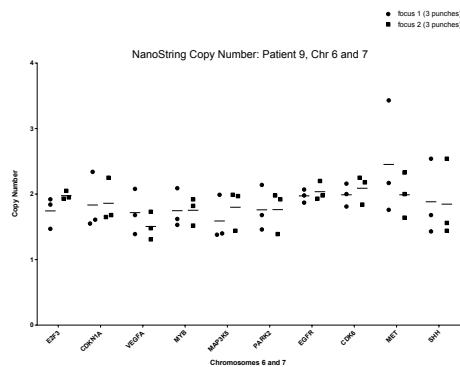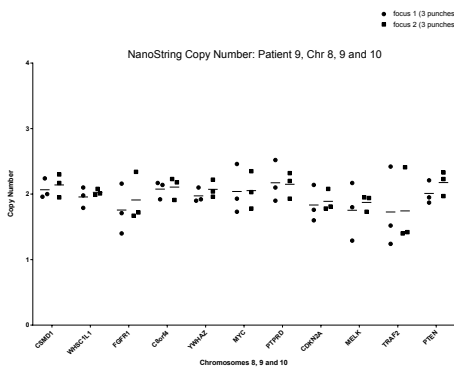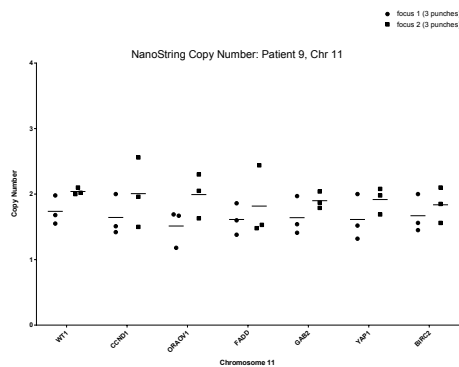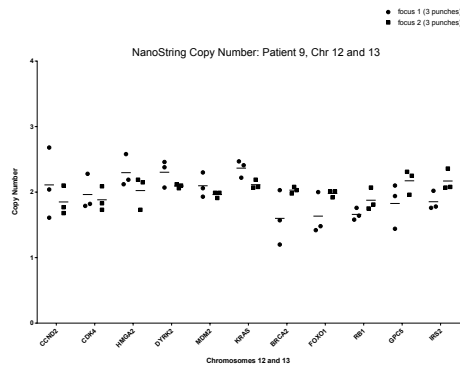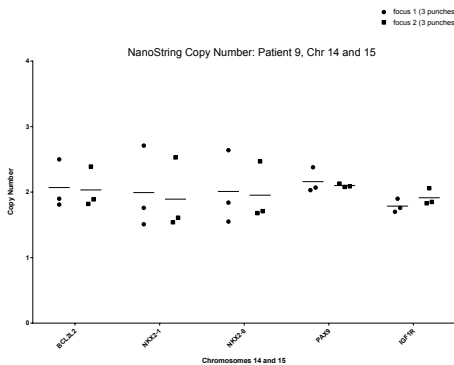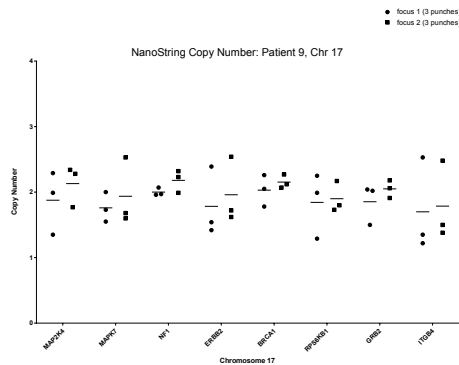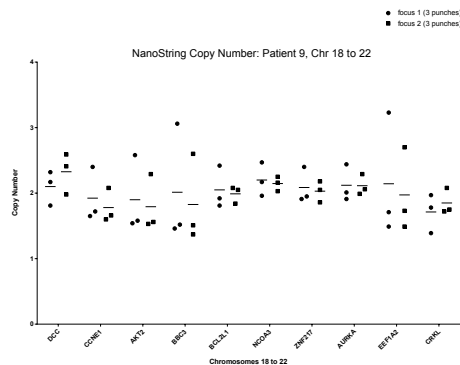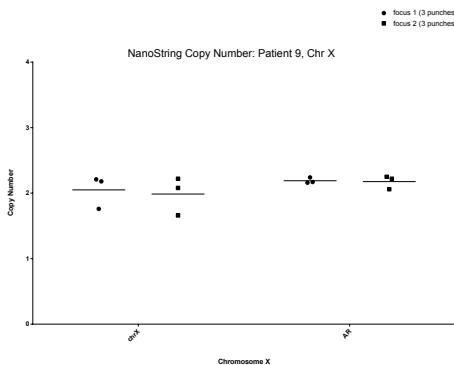

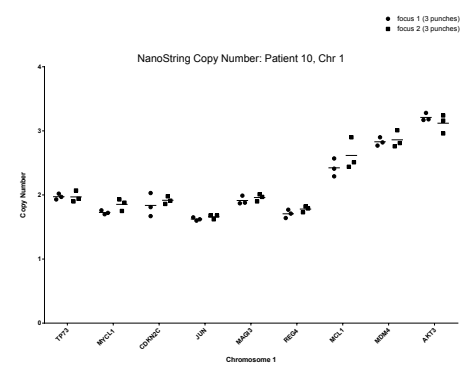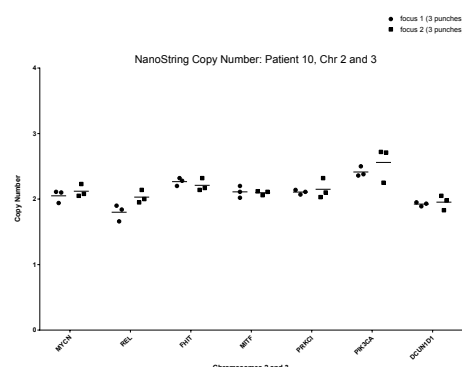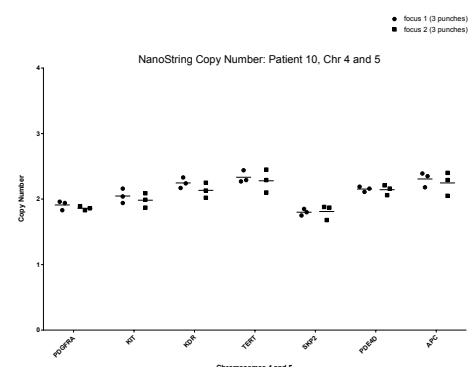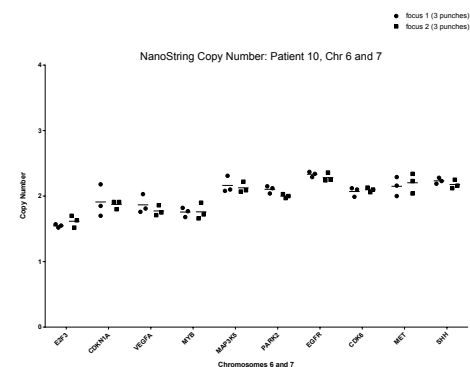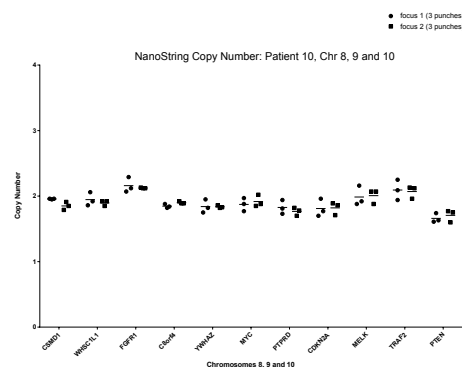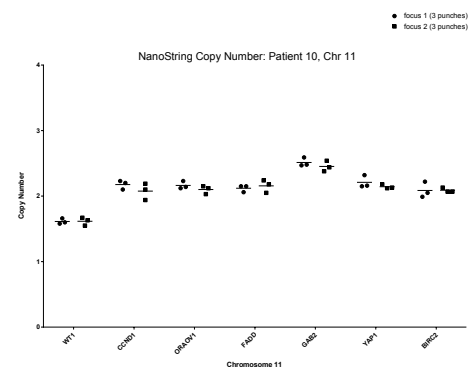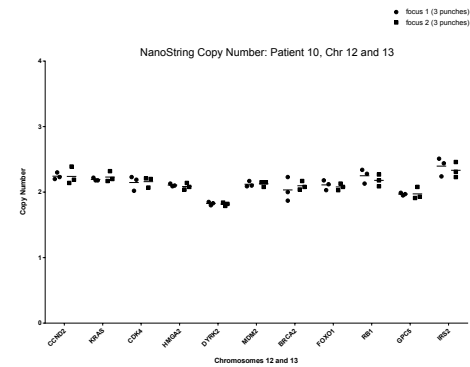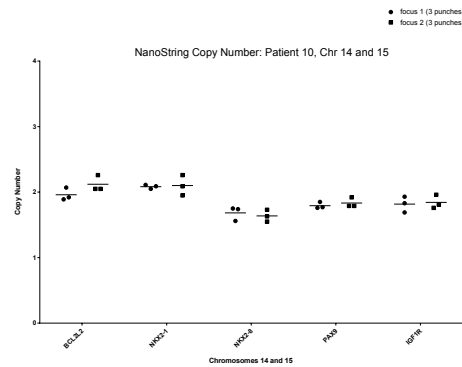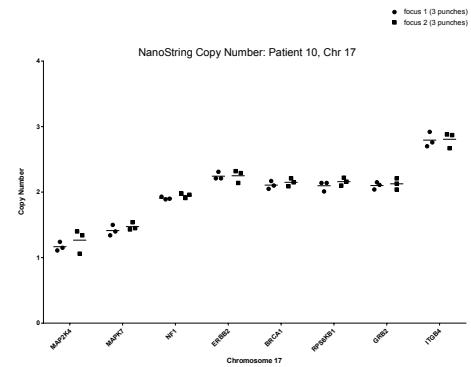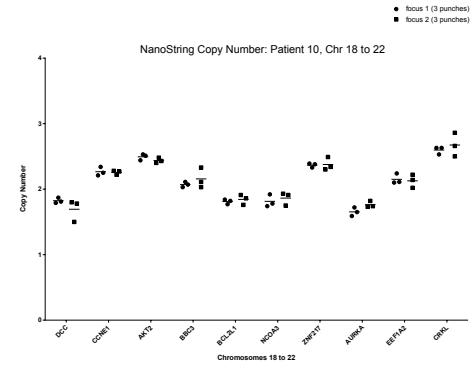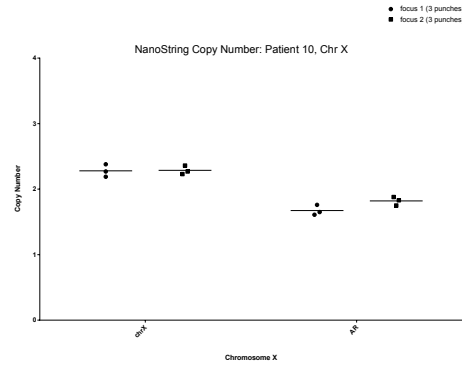

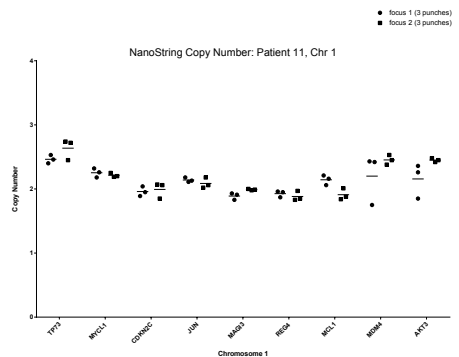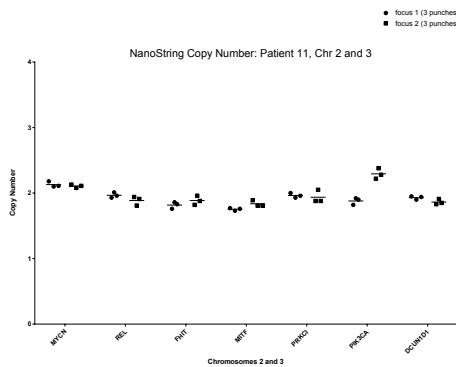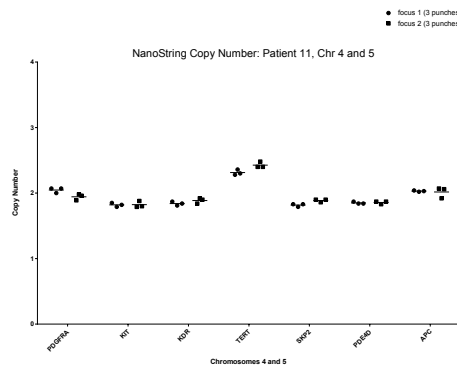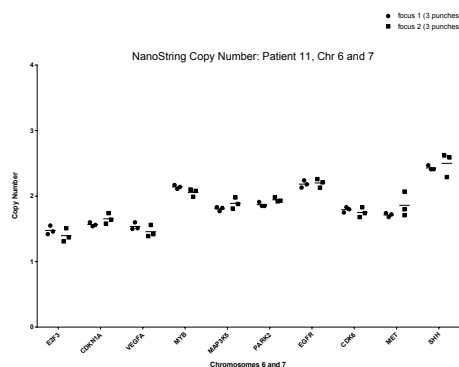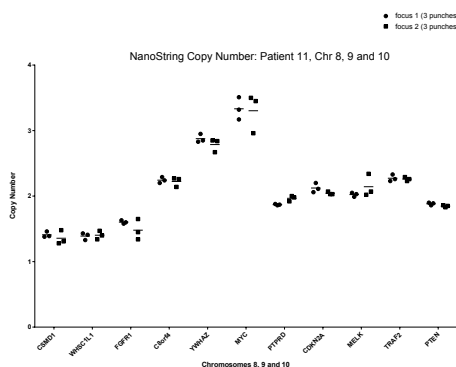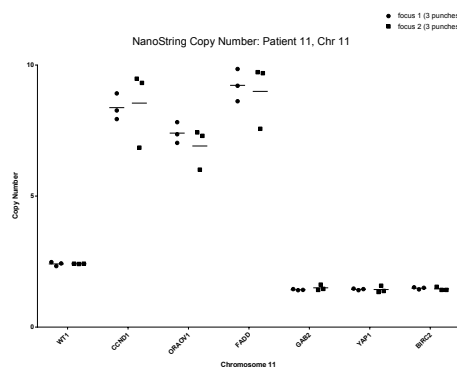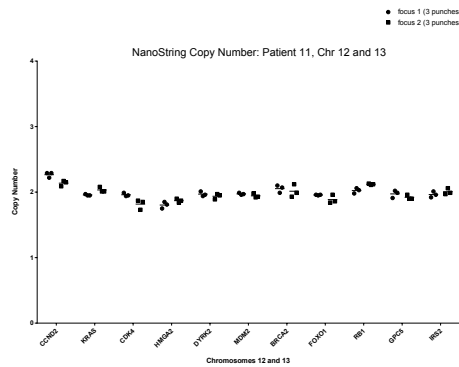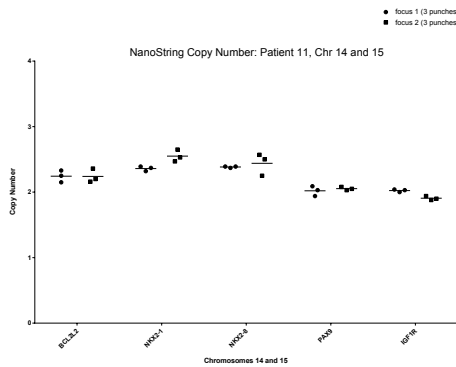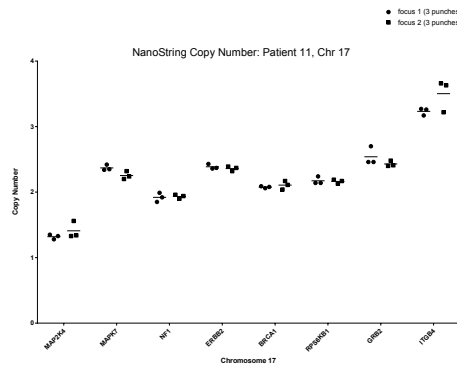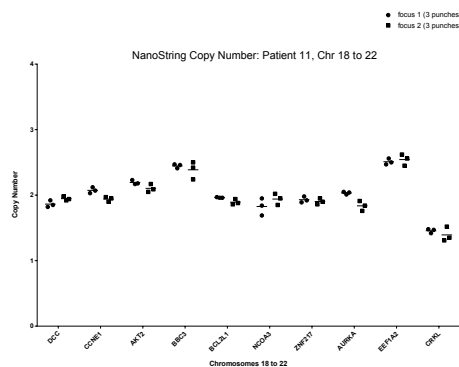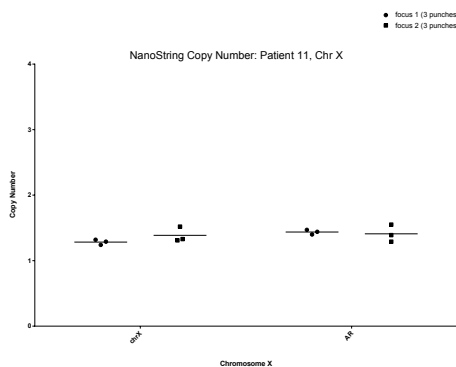

Supplement: S2 Fig — (PDF) [file pone.0153411.s002.pdf]

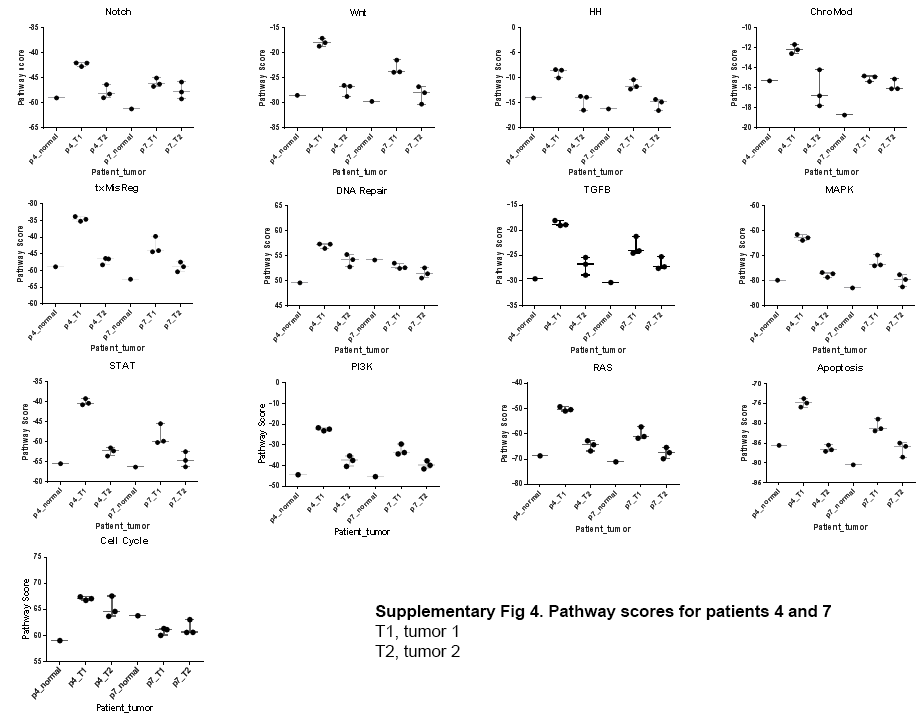

Supplement: S4 Fig — Each score from 3 core punches from each tumor and a single punch for adjacent normal tissue. Bars represent maximum, minimum and median score. (TIF) [file pone.0153411.s004.tif]
