# Supplementary material for: Assessment of Tumor Heterogeneity, as Evidenced by Gene Expression Profiles, Pathway Activation, and Gene Copy Number, in Patients with Multifocal Invasive Lobular Breast Tumors
Source: PLoS One. 2016 Apr 14;11(4):e0153411. doi: 10.1371/journal.pone.0153411 (PMC4831790; doi:10.1371/journal.pone.0153411)
Supplement: S3 Fig — Boxes represent maximum, minimum and mean score observed from 3x1.5mm core punches from each tumor and a single punch for adjacent normal tissue where available. Patients are labelled p1-p11 and tumors are labelled T1 and T2. Patient 1 has three tumors labelled p1_T1, p1_T2 and p1_T3. Patients 4 and 7 with the most differences in pathway score between T1 and T2 are highlighted in red and blue respectively. (PDF) [file pone.0153411.s003.pdf]

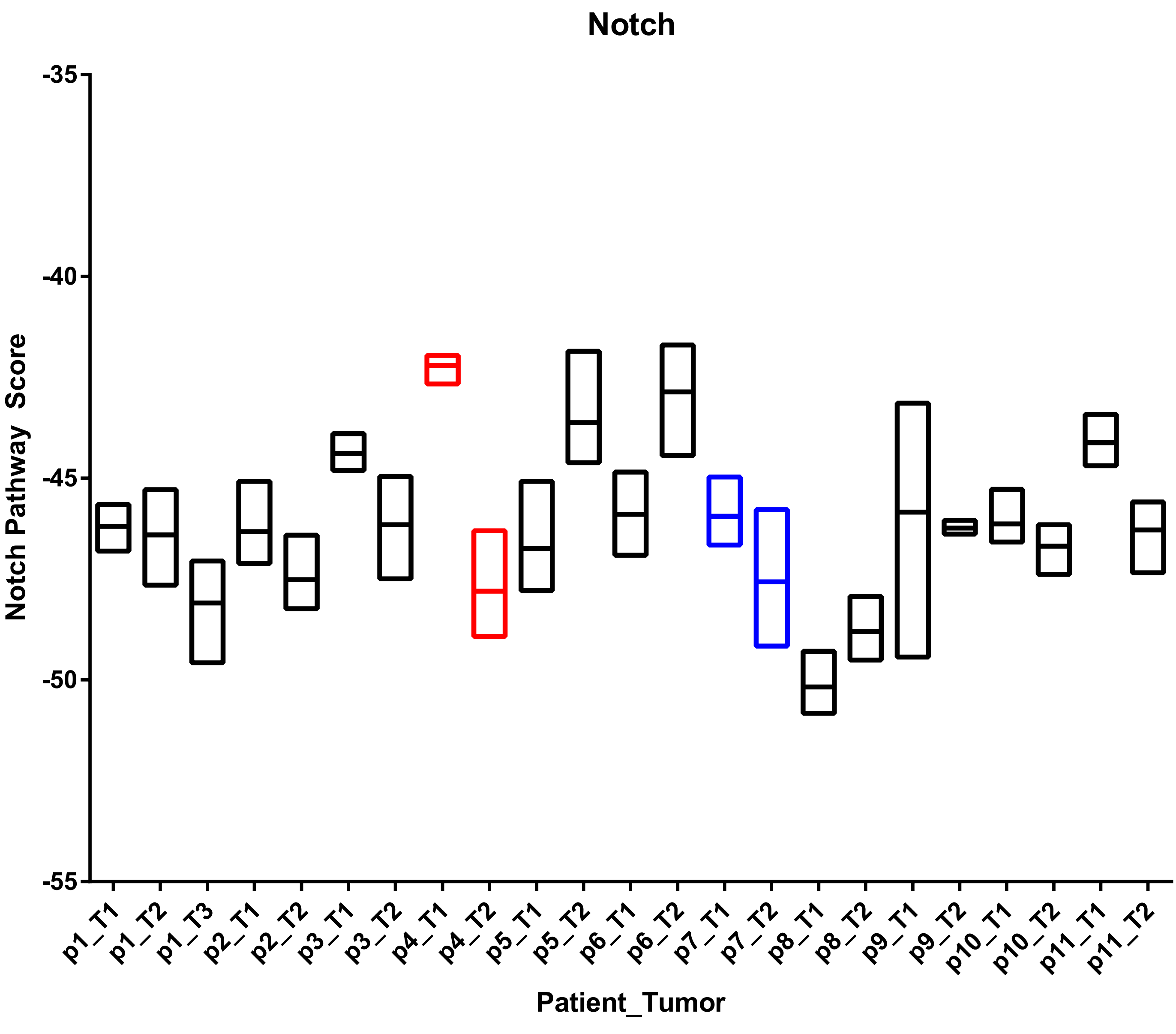
Notch
Notch Pathway Score
-35
-40
-45
-50
-55
p1_T1
p1_T2
p1_T3
p2_T1
p2_T2
p3_T1
p3_T2
p4_T1
p4_T2
p5_T1
p5_T2
p6_T1
p6_T2
p7_T1
p7_T2
p8_T1
p8_T2
p9_T1
p9_T2
p10_T1
p10_T2
p11_T1
p11_T2
Patient_Tumor

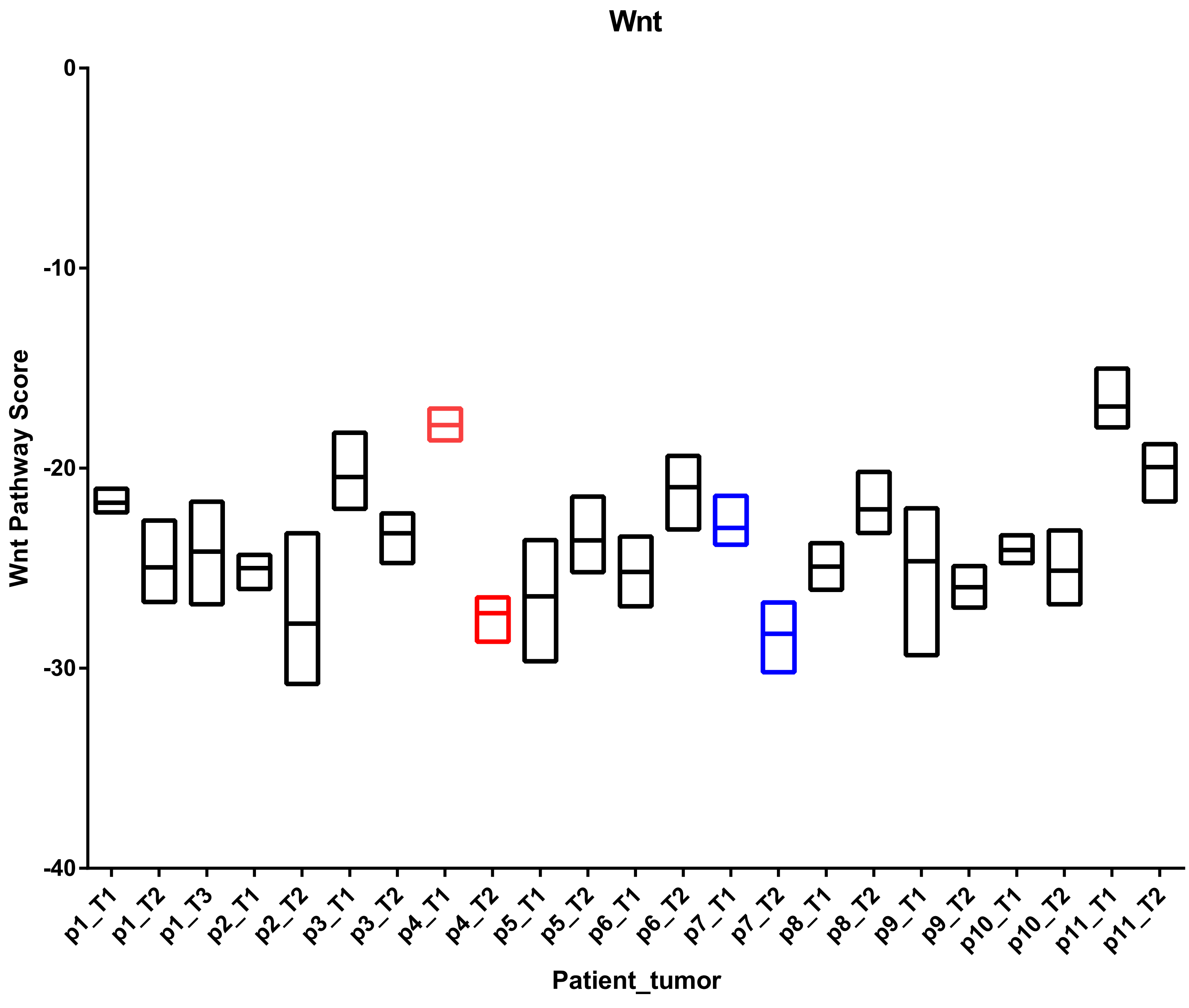

Wnt
Wnt Pathway Score
0
-10
-20
-30
-40
p1_T1
p1_T2
p1_T3
p2_T1
p2_T2
p3_T1
p3_T2
p4_T1
p4_T2
p5_T1
p5_T2
p6_T1
p6_T2
p7_T1
p7_T2
p8_T1
p8_T2
p9_T1
p9_T2
p10_T1
p10_T2
p11_T1
p11_T2
Patient_tumor

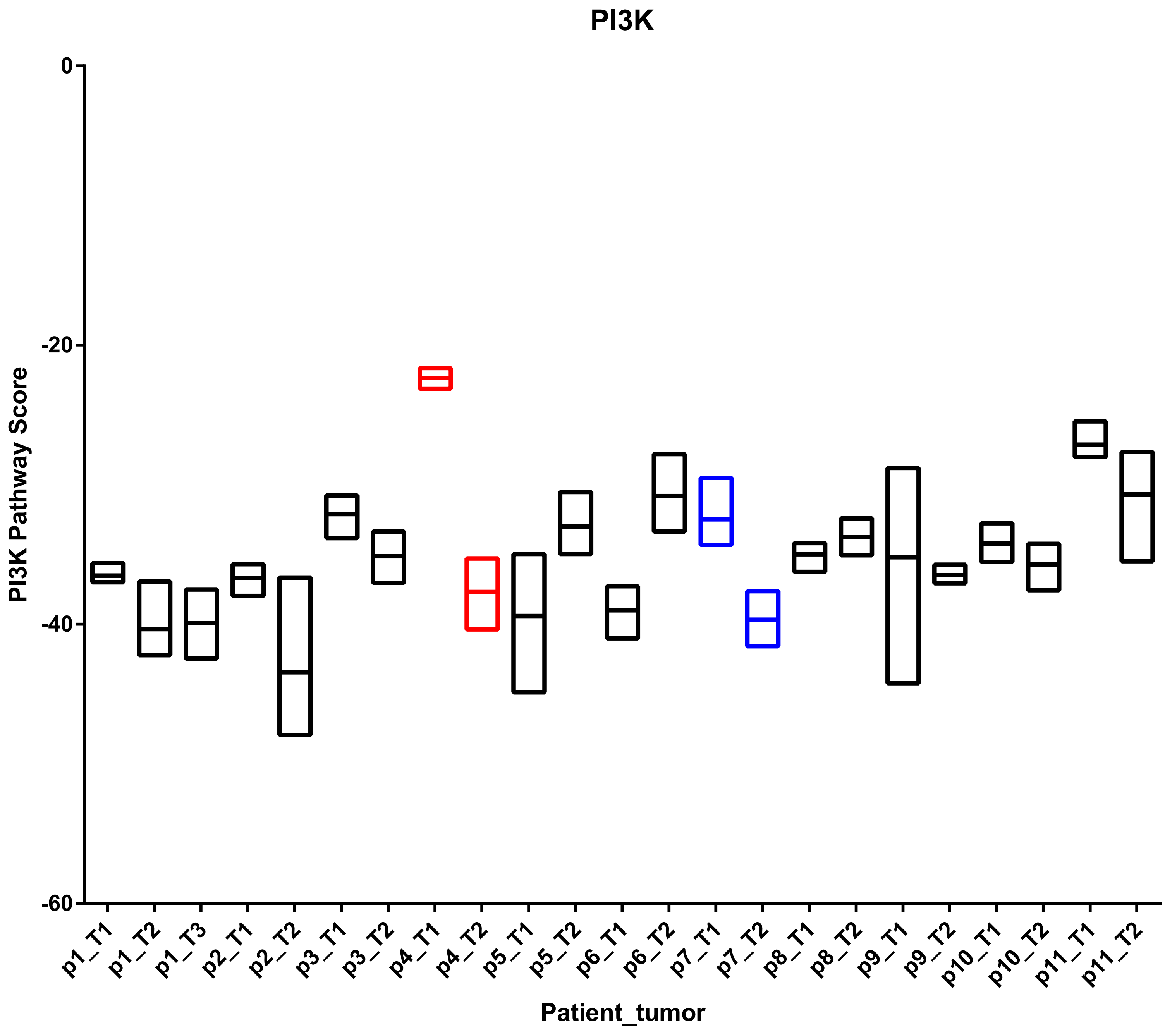
PI3K
0
-20
-40
-60
PI3K Pathway Score
p1_T1
p1_T2
p1_T3
p2_T1
p2_T2
p3_T1
p3_T2
p4_T1
p4_T2
p5_T1
p5_T2
p6_T1
p6_T2
p7_T1
p7_T2
p8_T1
p8_T2
p9_T1
p9_T2
p10_T1
p10_T2
p11_T1
p11_T2
Patient_tumor

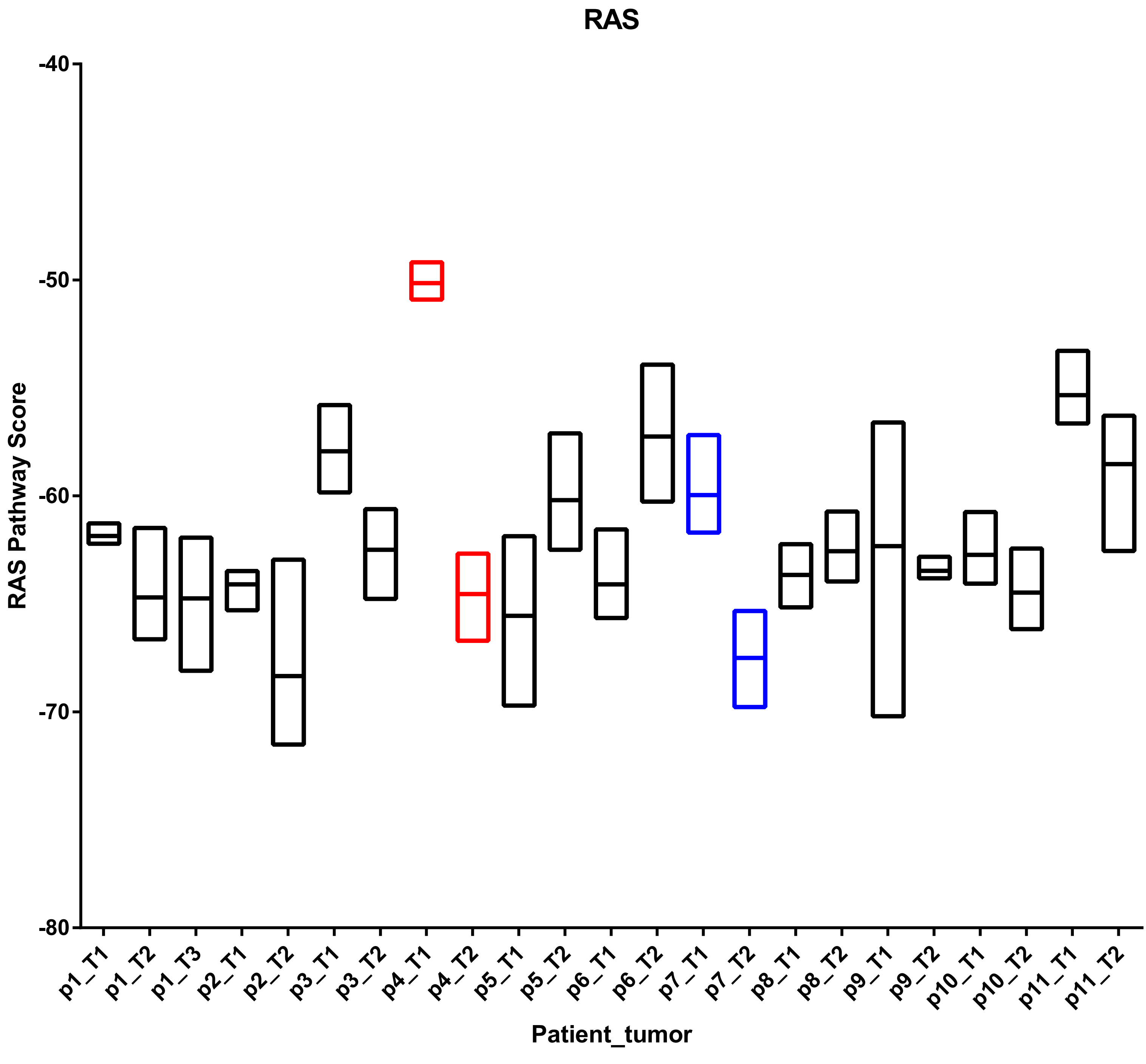
RAS
RAS Pathway Score
-40
-50
-60
-70
-80
p1_T1
p1_T2
p1_T3
p2_T1
p2_T2
p3_T1
p3_T2
p4_T1
p4_T2
p5_T1
p5_T2
p6_T1
p6_T2
p7_T1
p7_T2
p8_T1
p8_T2
p9_T1
p9_T2
p10_T1
p10_T2
p11_T1
p11_T2
Patient_tumor

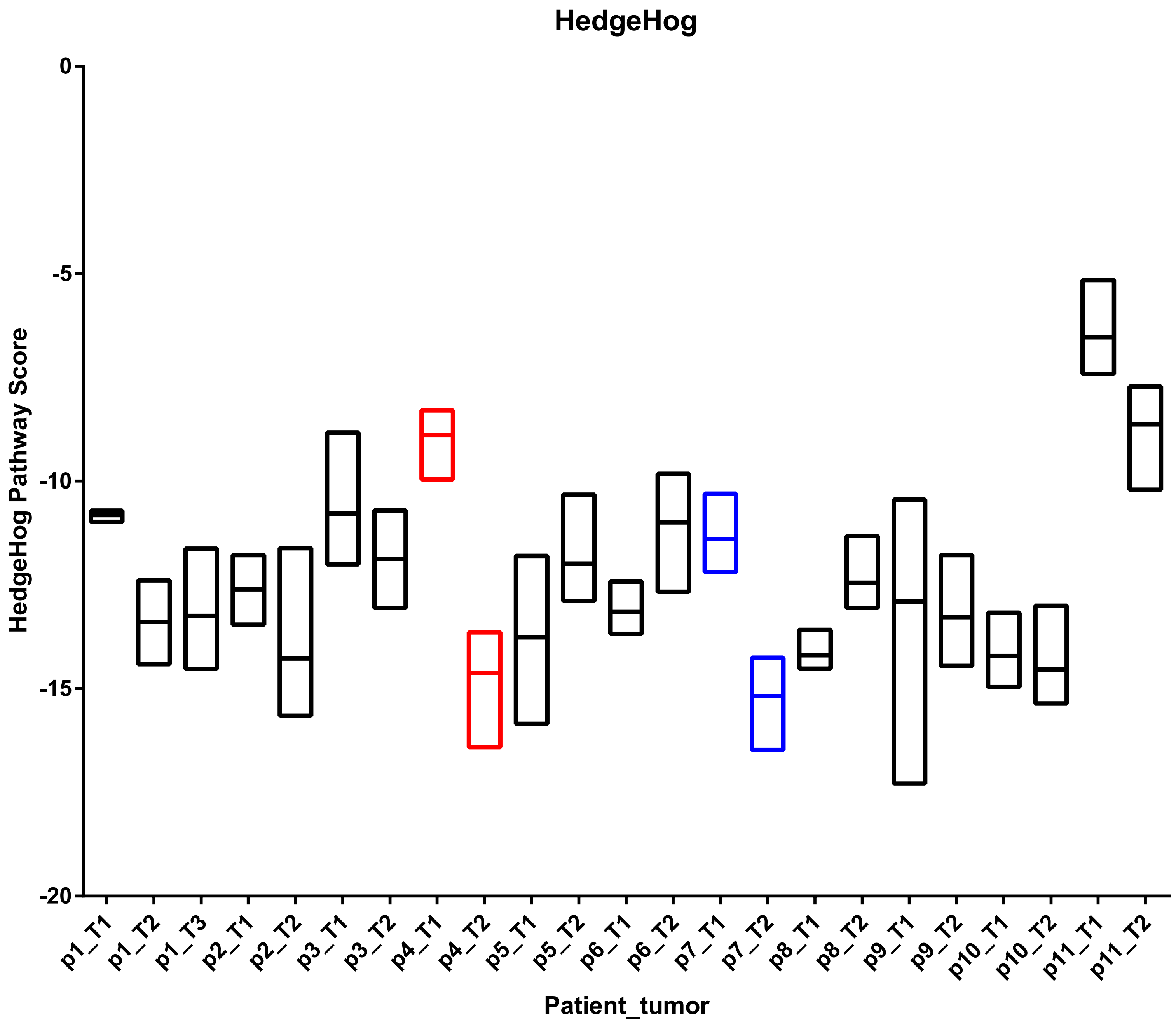
HedgeHog
HedgeHog Pathway Score
0
-5
-10
-15
-20
p1_T1
p1_T2
p1_T3
p2_T1
p2_T2
p3_T1
p3_T2
p4_T1
p4_T2
p5_T1
p5_T2
p6_T1
p6_T2
p7_T1
p7_T2
p8_T1
p8_T2
p9_T1
p9_T2
p10_T1
p10_T2
p11_T1
p11_T2
Patient_tumor

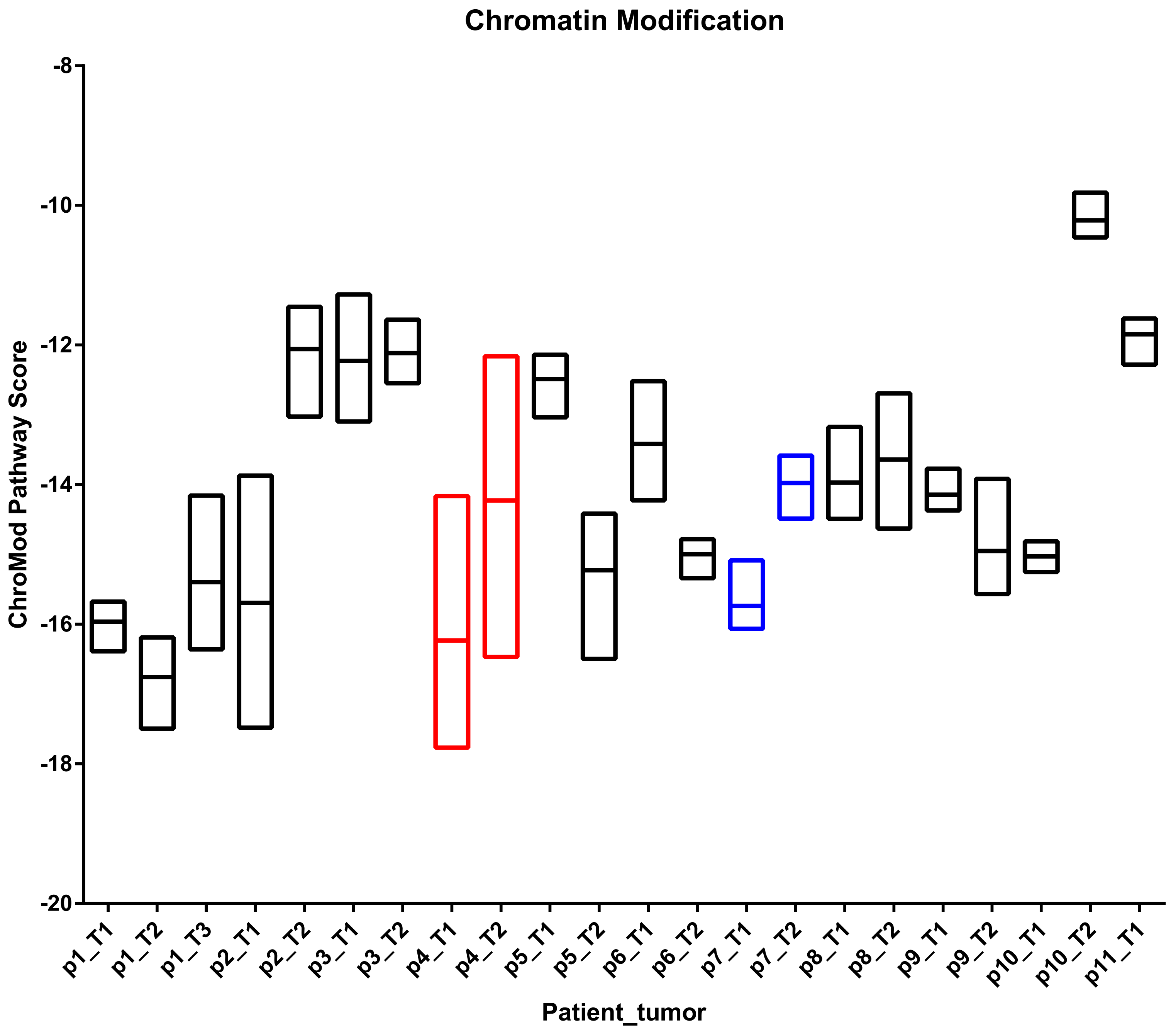

Chromatin Modification
ChroMod Pathway Score
-8
-10
-12
-14
-16
-18
-20
p1_T1
p1_T2
p1_T3
p2_T1
p2_T2
p3_T1
p3_T2
p4_T1
p4_T2
p5_T1
p5_T2
p6_T1
p6_T2
p7_T1
p7_T2
p8_T1
p8_T2
p9_T1
p9_T2
p10_T1
p10_T2
p11_T1
Patient_tumor

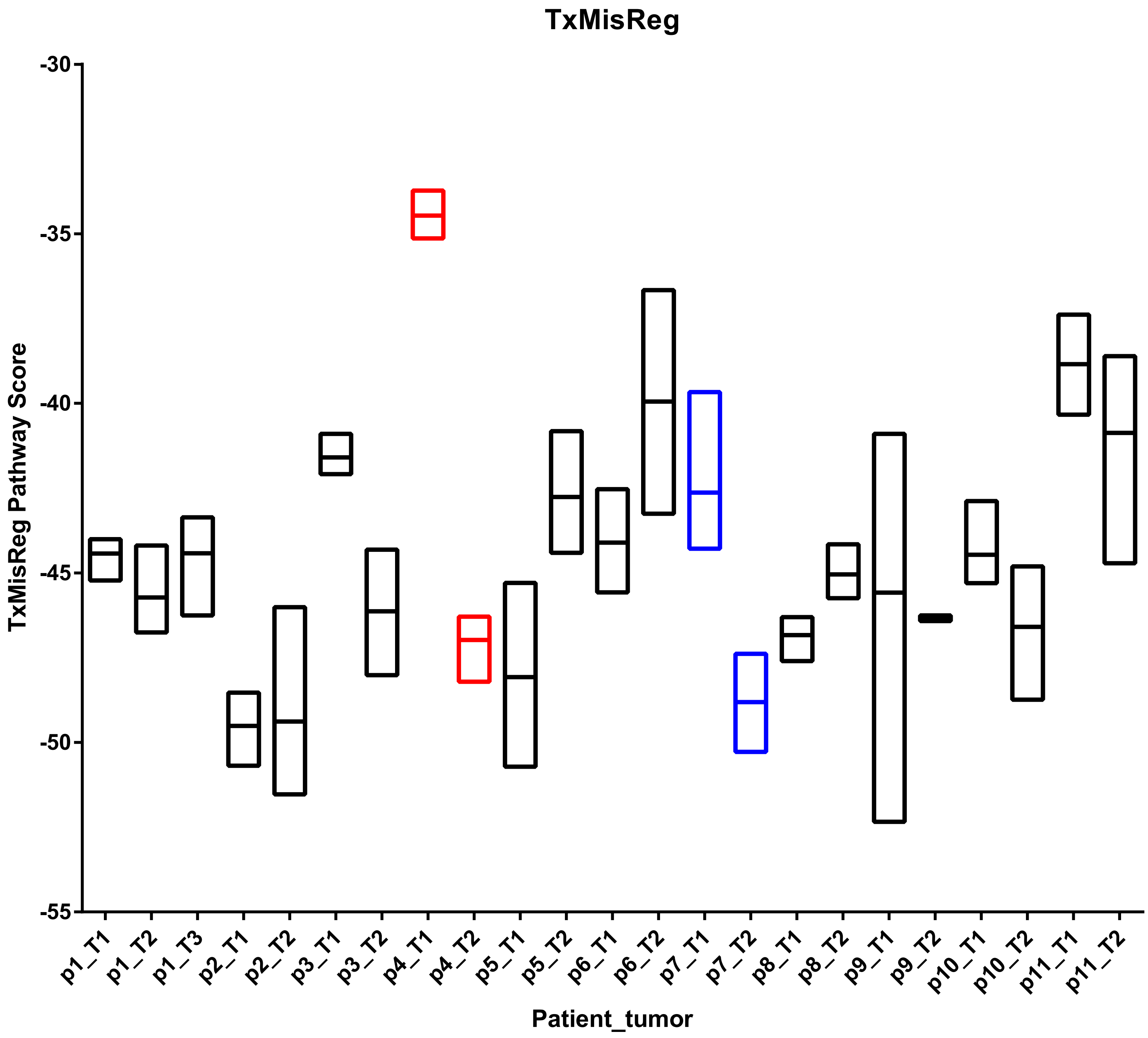
TxMisReg
TxMisReg Pathway Score
-30
-35
-40
-45
-50
-55
p1_T1
p1_T2
p1_T3
p2_T1
p2_T2
p3_T1
p3_T2
p4_T1
p4_T2
p5_T1
p5_T2
p6_T1
p6_T2
p7_T1
p7_T2
p8_T1
p8_T2
p9_T1
p9_T2
p10_T1
p10_T2
p11_T1
p11_T2
Patient_tumor

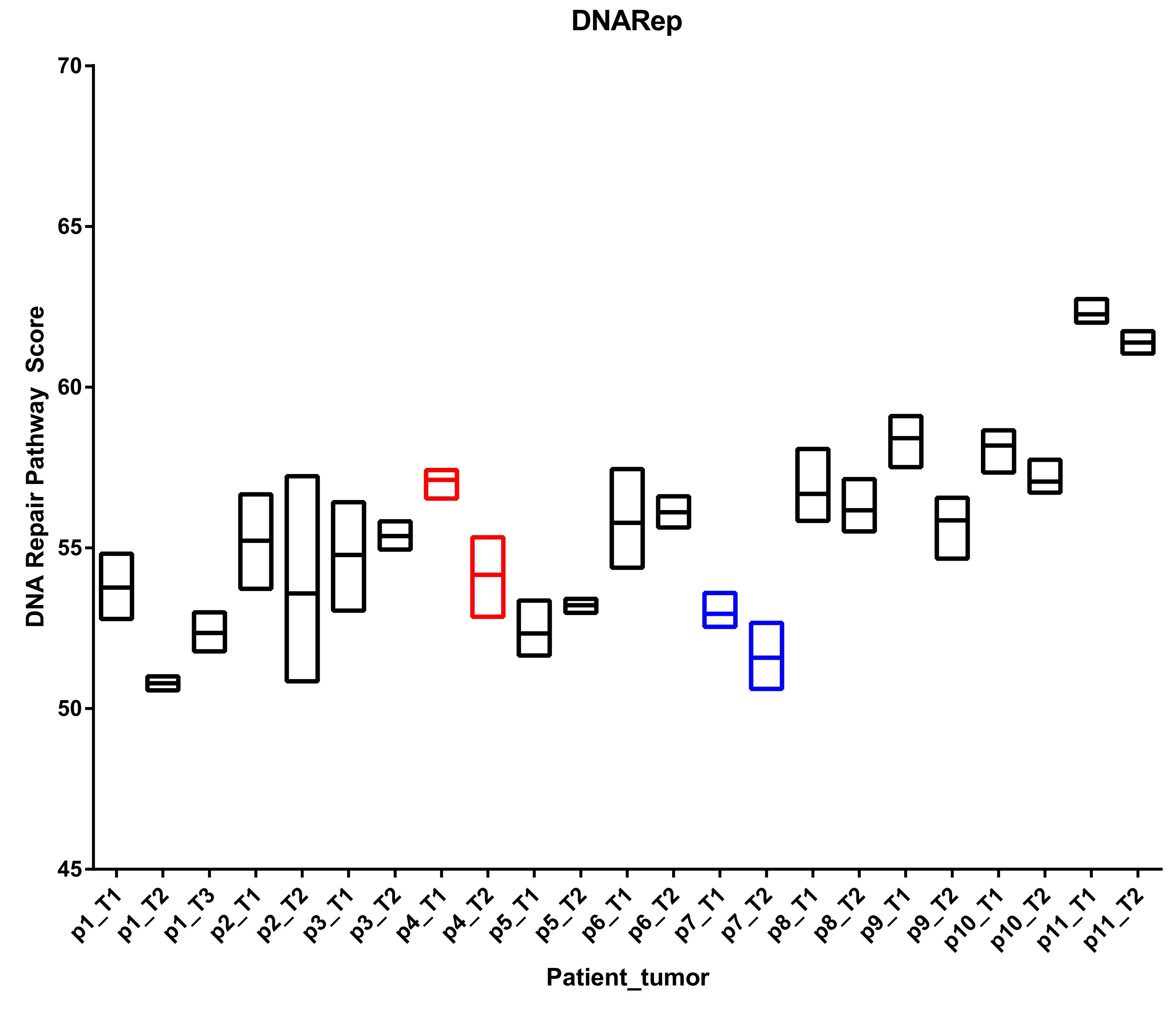
DNARep
DNA Repair Pathway Score
70
65
60
55
50
45
p1_T1
p1_T2
p1_T3
p2_T1
p2_T2
p3_T1
p3_T2
p4_T1
p4_T2
p5_T1
p5_T2
p6_T1
p6_T2
p7_T1
p7_T2
p8_T1
p8_T2
p9_T1
p9_T2
p10_T1
p10_T2
p11_T1
p11_T2
Patient_tumor

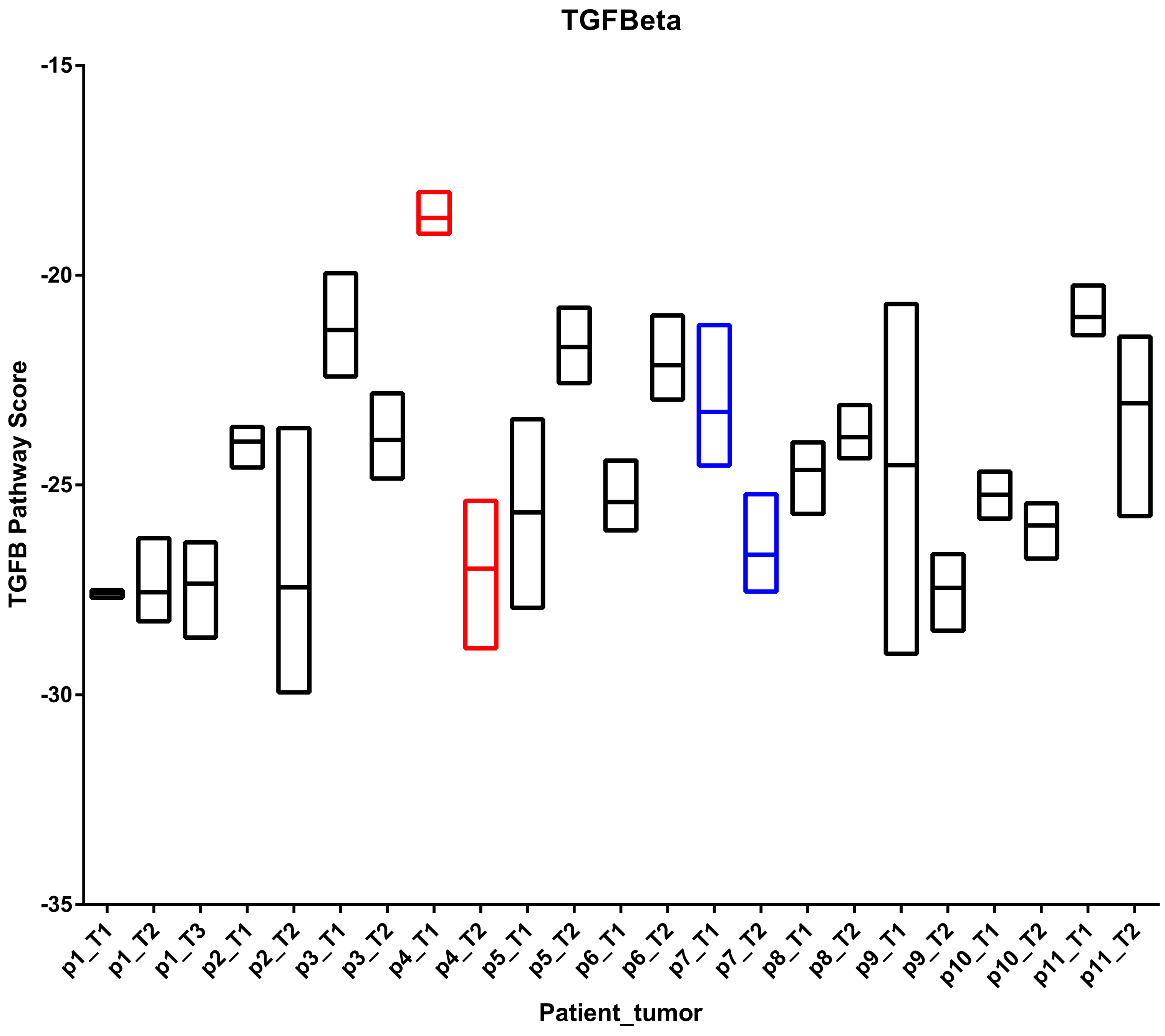

TGFBeta
TGFB Pathway Score
-15
-20
-25
-30
-35
p1_T1
p1_T2
p1_T3
p2_T1
p2_T2
p3_T1
p3_T2
p4_T1
p4_T2
p5_T1
p5_T2
p6_T1
p6_T2
p7_T1
p7_T2
p8_T1
p8_T2
p9_T1
p9_T2
p10_T1
p10_T2
p11_T1
p11_T2
Patient_tumor

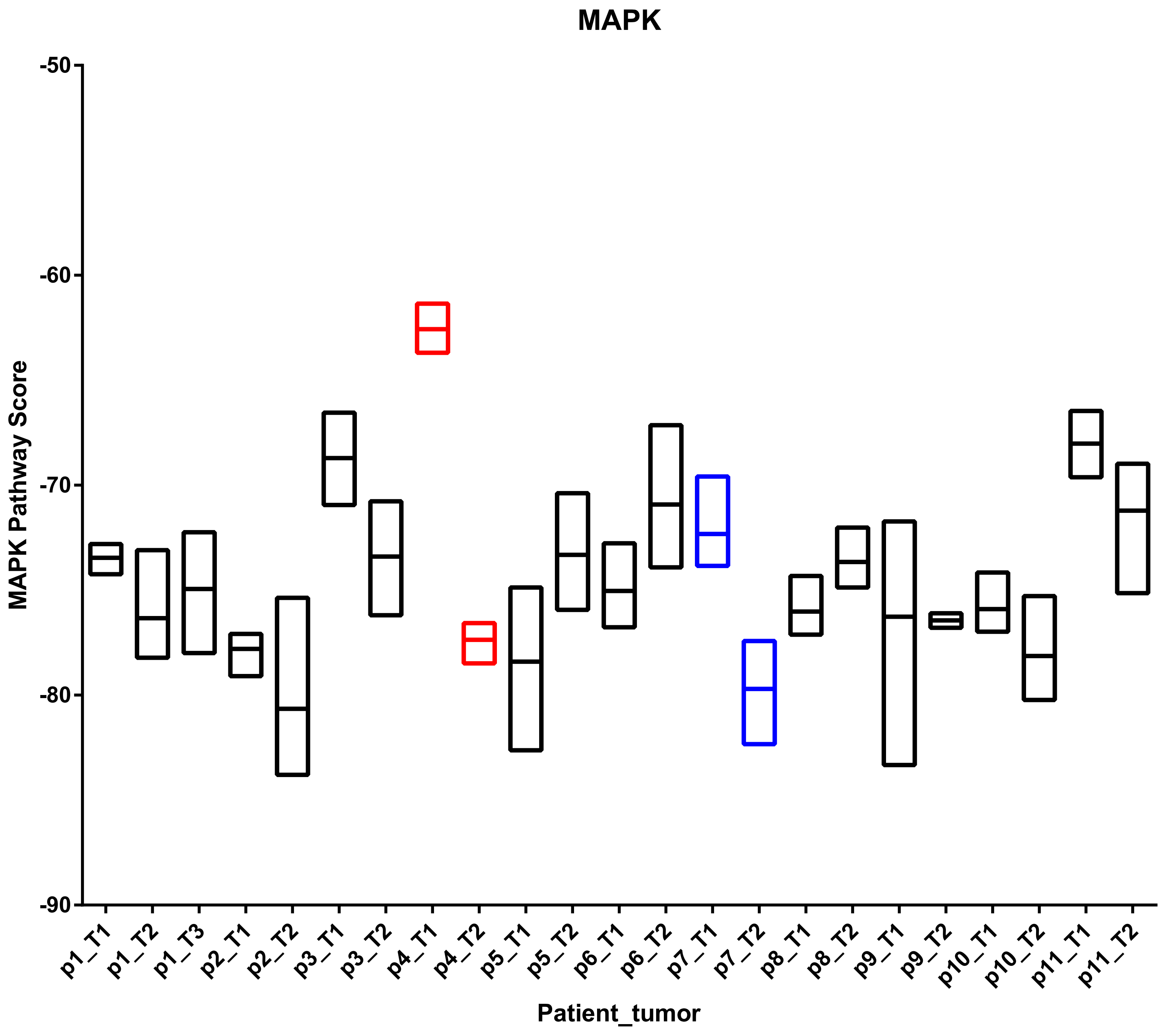
MAPK
MAPK Pathway Score
-50
-60
-70
-80
-90
p1_T1
p1_T2
p1_T3
p2_T1
p2_T2
p3_T1
p3_T2
p4_T1
p4_T2
p5_T1
p5_T2
p6_T1
p6_T2
p7_T1
p7_T2
p8_T1
p8_T2
p9_T1
p9_T2
p10_T1
p10_T2
p11_T1
p11_T2
Patient_tumor

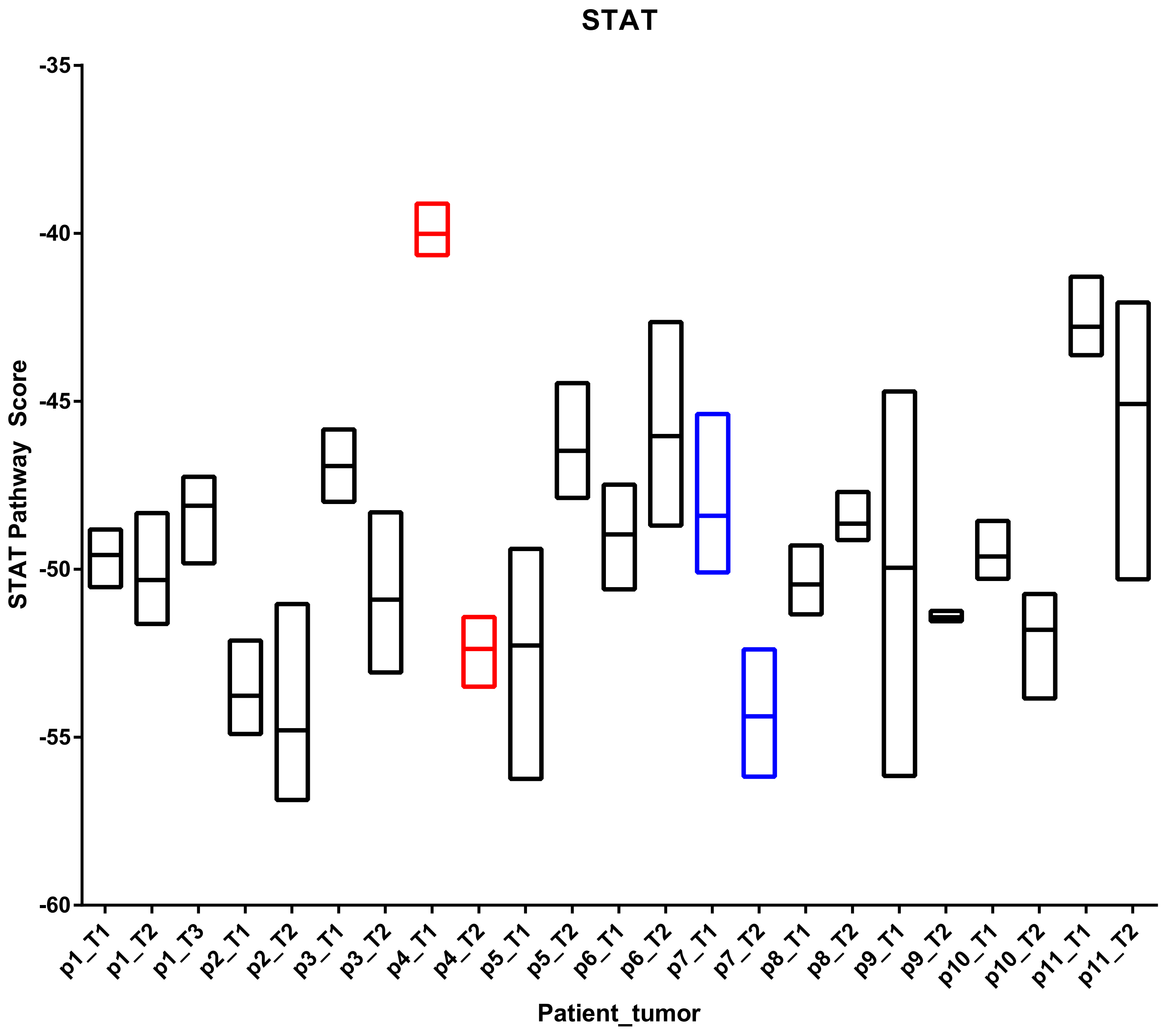

STAT
STAT Pathway Score
-35
-40
-45
-50
-55
-60
p1_T1
p1_T2
p1_T3
p2_T1
p2_T2
p3_T1
p3_T2
p4_T1
p4_T2
p5_T1
p5_T2
p6_T1
p6_T2
p7_T1
p7_T2
p8_T1
p8_T2
p9_T1
p9_T2
p10_T1
p10_T2
p11_T1
p11_T2
Patient_tumor

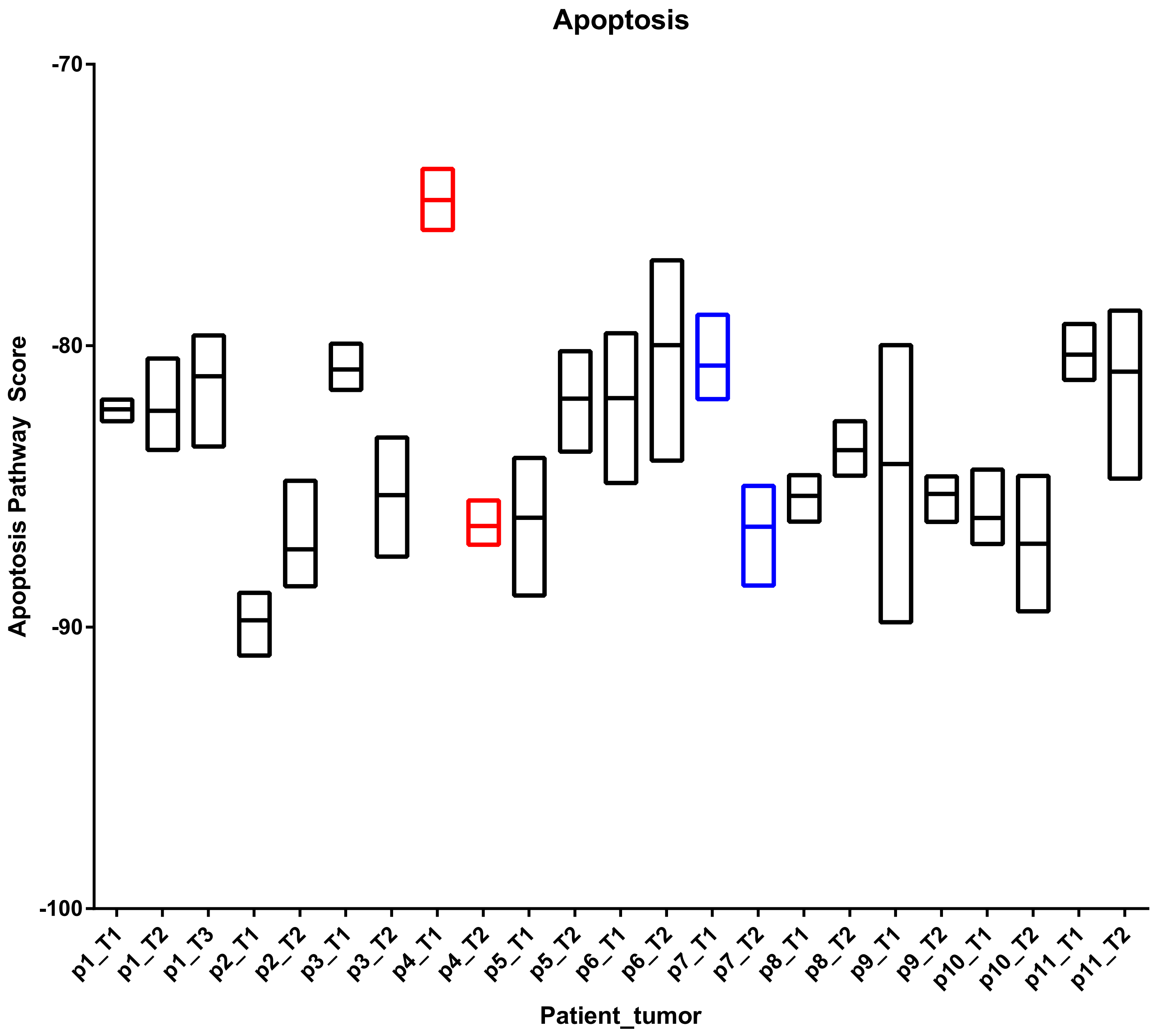

Apoptosis
Apoptosis Pathway Score
-70
-80
-90
-100
p1_T1
p1_T2
p1_T3
p2_T1
p2_T2
p3_T1
p3_T2
p4_T1
p4_T2
p5_T1
p5_T2
p6_T1
p6_T2
p7_T1
p7_T2
p8_T1
p8_T2
p9_T1
p9_T2
p10_T1
p10_T2
p11_T1
p11_T2
Patient_tumor

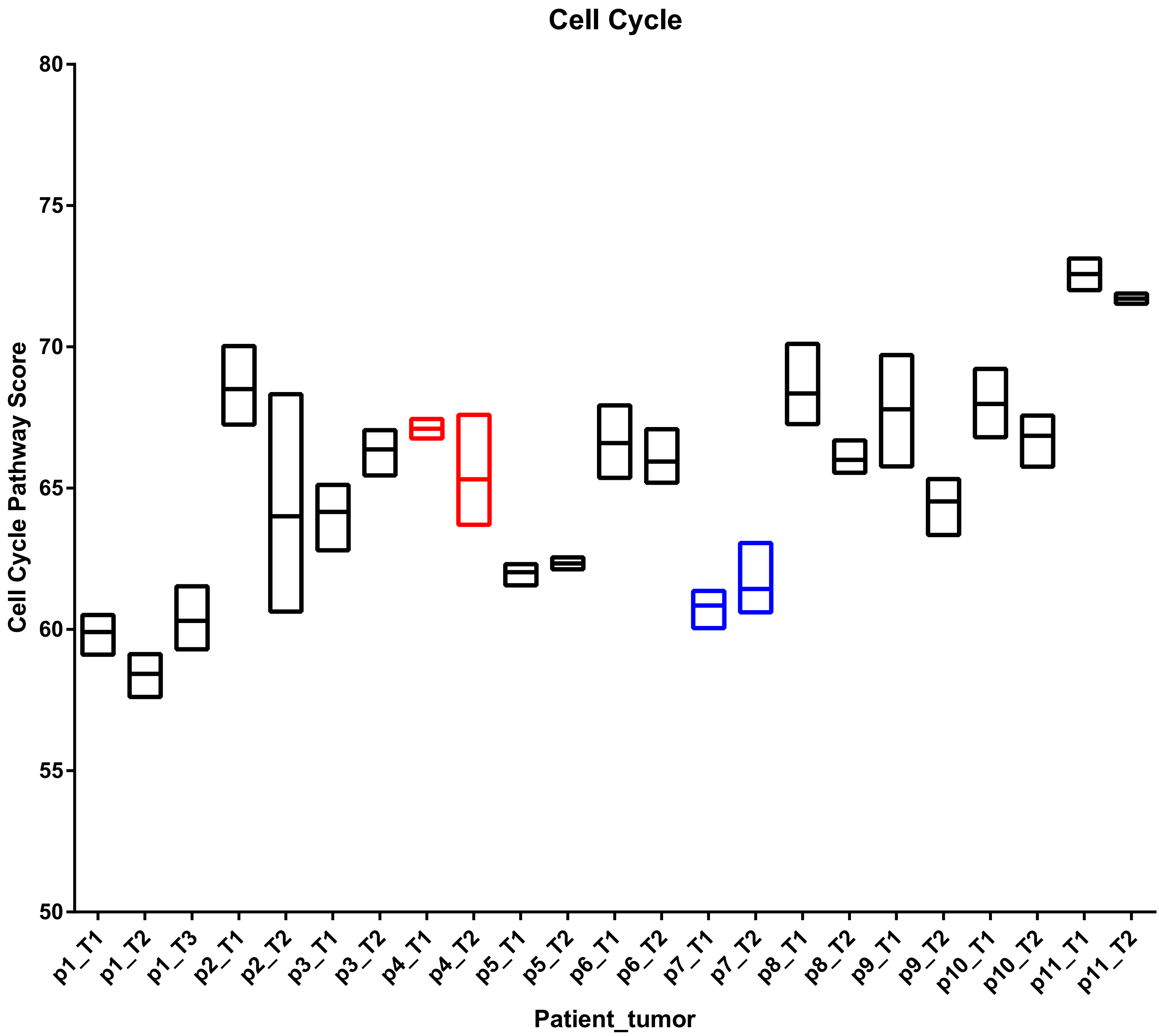

Cell Cycle
Cell Cycle Pathway Score
80
75
70
65
60
55
50
p1_T1
p1_T2
p1_T3
p2_T1
p2_T2
p3_T1
p3_T2
p4_T1
p4_T2
p5_T1
p5_T2
p6_T1
p6_T2
p7_T1
p7_T2
p8_T1
p8_T2
p9_T1
p9_T2
p10_T1
p10_T2
p11_T1
p11_T2
Patient_tumor
