# Supplementary material for: Assessment of Tumor Heterogeneity, as Evidenced by Gene Expression Profiles, Pathway Activation, and Gene Copy Number, in Patients with Multifocal Invasive Lobular Breast Tumors
Source: PLoS One. 2016 Apr 14;11(4):e0153411. doi: 10.1371/journal.pone.0153411 (PMC4831790; doi:10.1371/journal.pone.0153411)
Supplement: S2 Table — (DOCX) [file pone.0153411.s006.docx]

| **Pathway** | **Pathway Tumor vs normal Log2 fold change** | **Pathway Tumor vs normal absolute fold change** | **Pathway t statistic** | **Pathway P-value** | **Global significance statistic** |
| --- | --- | --- | --- | --- | --- |
| Apop | 3.314 | 9.944 | 2.564 | 0.0127013 | 2.109 |
| CC | 4.541 | 23.281 | 5.233 | 0.0000020 | 2.848 |
| ChromMod | 2.167 | 4.491 | 3.913 | 0.0002234 | 2.130 |
| DNARepair | 3.732 | 13.284 | 5.180 | 0.0000024 | 2.628 |
| HH | 2.624 | 6.163 | 3.310 | 0.0015336 | 1.918 |
| MAPK | 7.461 | 176.149 | 4.534 | 0.0000260 | 2.411 |
| Notch | 2.727 | 6.621 | 3.901 | 0.0002326 | 2.425 |
| PI3K | 10.100 | 1097.659 | 5.610 | 0.0000005 | 2.710 |
| RAS | 8.185 | 290.940 | 4.881 | 0.0000074 | 2.496 |
| STAT | 5.249 | 38.029 | 3.812 | 0.0003122 | 2.374 |
| TGFB | 4.756 | 27.027 | 4.957 | 0.0000056 | 2.523 |
| TXmisReg | 5.142 | 35.312 | 3.792 | 0.0003339 | 2.350 |
| Wnt | 5.487 | 44.847 | 4.712 | 0.0000137 | 2.448 |

**Supplementary Table 2. Differential Pathway Expression between ILC and adjacent normal tissue.**
