# Supplementary material for: Assessment of Tumor Heterogeneity, as Evidenced by Gene Expression Profiles, Pathway Activation, and Gene Copy Number, in Patients with Multifocal Invasive Lobular Breast Tumors
Source: PLoS One. 2016 Apr 14;11(4):e0153411. doi: 10.1371/journal.pone.0153411 (PMC4831790; doi:10.1371/journal.pone.0153411)
Supplement: S3 Table — (DOCX) [file pone.0153411.s007.docx]

| **Pathway** | **Pathway smaller vs larger tumor: log2 fold change** | **Pathway smaller vs larger tumor: absolute fold change** | **Pathway t statistic** | **Pathway P-value** | **Global significance statistic** |
| --- | --- | --- | --- | --- | --- |
| Apop | -1.287 | 0.410 | -1.714 | 0.092 | 1.964 |
| CC | -1.175 | 0.443 | -3.040 | 0.004 | 1.884 |
| ChromMod | -0.358 | 0.780 | -1.206 | 0.233 | 2.138 |
| DNARepair | -1.109 | 0.464 | -3.574 | 0.001 | 1.542 |
| HH | -1.088 | 0.470 | -2.362 | 0.022 | 2.279 |
| MAPK | -2.421 | 0.187 | -2.568 | 0.013 | 1.994 |
| Notch | -0.542 | 0.687 | -1.288 | 0.203 | 1.867 |
| PI3K | -2.421 | 0.187 | -2.246 | 0.029 | 1.951 |
| RAS | -2.398 | 0.190 | -2.453 | 0.017 | 1.962 |
| STAT | -1.773 | 0.293 | -2.173 | 0.034 | 1.935 |
| TGFB | -1.415 | 0.375 | -2.464 | 0.017 | 2.117 |
| TXmisReg | -1.643 | 0.320 | -2.034 | 0.047 | 2.065 |
| Wnt | -1.726 | 0.302 | -2.566 | 0.013 | 2.185 |

**Supplementary table 4. Differential Pathway Expression in multiple foci within ILC patients.**
